# HvAKT2 and HvHAK1 confer drought tolerance in barley through enhanced leaf mesophyll H^+^ homoeostasis

**DOI:** 10.1111/pbi.13332

**Published:** 2020-01-24

**Authors:** Xue Feng, Wenxing Liu, Cheng‐Wei Qiu, Fanrong Zeng, Yizhou Wang, Guoping Zhang, Zhong‐Hua Chen, Feibo Wu

**Affiliations:** ^1^ Department of Agronomy College of Agriculture and Biotechnology Zhejiang University Hangzhou China; ^2^ Jiangsu Co‐Innovation Center for Modern Production Technology of Grain Crops Yangzhou University Yangzhou China; ^3^ School of Science Hawkesbury Institute for the Environment Western Sydney University Penrith NSW Australia; ^4^ Collaborative Innovation Center for Grain Industry College of Agriculture Yangtze University Jingzhou China

**Keywords:** drought adaptation, ion fluxes, K^+^ channel, K^+^ transporter, wild barley

## Abstract

Plant K^+^ uptake typically consists low—affinity mechanisms mediated by Shaker K^+^ channels (AKT/KAT/KC) and high‐affinity mechanisms regulated by HAK/KUP/KT transporters, which are extensively studied. However, the evolutionary and genetic roles of both K^+^ uptake mechanisms for drought tolerance are not fully explored in crops adapted to dryland agriculture. Here, we employed evolutionary bioinformatics, biotechnological and electrophysiological approaches to determine the role of two important K^+^ transporters HvAKT2 and HvHAK1 in drought tolerance in barley. *HvAKT2* and *HvHAK1* were cloned and functionally characterized using barley stripe mosaic virus‐induced gene silencing (BSMV‐VIGS) in drought‐tolerant wild barley XZ5 and agrobacterium‐mediated gene transfer in the barley cultivar Golden Promise. The hallmarks of the K^+^ selective filters of AKT2 and HAK1 are both found in homologues from strepotophyte algae, and they are evolutionarily conserved in strepotophyte algae and land plants. HvAKT2 and HvHAK1 are both localized to the plasma membrane and have high selectivity to K^+^ and Rb^+^ over other tested cations. Overexpression of *HvAKT2* and *HvHAK1* enhanced K^+^ uptake and H^+^ homoeostasis leading to drought tolerance in these transgenic lines. Moreover, *HvAKT2‐* and *HvHAK1‐*overexpressing lines showed distinct response of K^+^, H^+^ and Ca^2+^ fluxes across plasma membrane and production of nitric oxide and hydrogen peroxide in leaves as compared to the wild type and silenced lines. High‐ and low‐affinity K^+^ uptake mechanisms and their coordination with H^+^ homoeostasis play essential roles in drought adaptation of wild barley. These findings can potentially facilitate future breeding programs for resilient cereal crops in a changing global climate.

## Introduction

Severe drought can affect terrestrial ecosystems at regional to global scales, and the intensity and duration of drought significantly affect plant productivity and the health of ecosystems. Hence, understanding drought tolerance mechanisms in crops is crucial (Li *et al.*, 2017; Selvaraj *et al.*, 2017; Umezawa *et al.*, 2006). Potassium (K^+^) is the most abundant inorganic essential cation in plants and contributes up to 10% of their dry mass (Marschner, 2012). In plants, K^+^ is involved in enzyme function, the maintenance of turgor pressure, leaf, and stomatal movement, and cell elongation, playing important roles under abiotic stresses (Dreyer and Uozumi, 2011). Drought tolerance in plants has been linked to K^+^ homoeostasis (Mak *et al.*, 2014; Shabala and Pottosin, 2014; Zhang *et al.*, 2019).

During the early evolution of life, K^+^ was utilized by cells as the major cation to maintain electroneutrality and osmotic equilibrium. Land plants have evolved from streptophyte algae and further evolution of biochemical processes, resulting in K^+^ to be an absolutely necessary element (Rodriguez‐Navarro and Rubio, 2006; Zhao *et al.*, 2019). The emergence of the terrestrial plants in the Cambrian era and their evolution from bryophytes to flowering plants took place in limited K^+^ conditions and periodical drought events (Heckman *et al.*, 2001; Qiu and Palmer, 1999; Rodriguez‐Navarro and Rubio, 2006). Despite the lack of K^+^, terrestrial plants not only kept their K^+^ dependence but also developed new functions for efficient utilization of K^+^ (Chen *et al.*, 2017; Rodriguez‐Navarro and Rubio, 2006). Therefore, the terrestrial plants have evolved high‐ and low‐affinity K^+^ uptake systems with multiple K^+^ transport families for K^+^ uptake and translocation to various plant tissues to respond to changing environmental conditions (Ahn, 2004; Shabala and Cuin, 2008; Marschner, 2012; Cai *et al*., 2017; Zhao *et al.*, 2019).

The classic high‐ and low‐affinity K^+^ uptake mechanisms in plants (Epstein *et al.*, 1963) have been validated through functionally characterization of key transporters mainly in model plants (Dreyer and Uozumi, 2011; Rodriguez‐Navarro and Rubio, 2006). The transport of K^+^ through the plasma membrane into the plant and its allocation within the plant are mediated by K^+^ transporters (HAK/KUP/KTs, HKTs), K^+^/H^+^ antiporters (NHXs) and Shaker‐type K^+^ channels (AKTs, KATs, KC1, GORK, SKOR) (Grabov, 2007; Dreyer and Uozumi, 2011; Chen *et al.*, 2016; Chen *et al.*, 2017). The role of many K^+^ transporters has been extensively studied (Chérel *et al.*, 2014; Véry and Sentenac, 2003). For example, the roles of K^+^ channels in stomata operation and abscisic acid (ABA) responses (Chen *et al.*, 2016; Hosy *et al.*, 2003) and increased root K^+^ uptake in osmotic stress (Fulgenzi *et al.*, 2008; Shabala and Lew, 2002) were demonstrated, but this link has not been fully validated at molecular level using gene silencing and overexpression in a non‐model plant. Also, the function of K^+^ transport‐related genes in barley such as *HvAKT2* and *HvHAK1* has not yet been fully elucidated and studies using gene‐silencing and gene‐overexpressing mutants in barley under drought stress need to be made.

The potassium channel AKT2, a component of low‐affinity K^+^ uptake system, plays important roles in long distance phloem loading and unloading of K^+^. It can operate as an inward‐rectifying channel that allows H^+^‐ATPase‐energized K^+^ uptake. AKT2 is also an important contributor, along with AKT1, to the mesophyll K^+^ permeability (Boscari *et al.*, 2009; Lacombe *et al.*, 2000; Sklodowski *et al.*, 2017; Véry and Sentenac, 2003). *Arabidopsis* AKT2 is strongly up‐regulated by ABA, implicating its potential role in drought tolerance (Lacombe *et al.*, 2000). When expressed in *Xenopus laevis* oocytes, HEK293 cells and COS cells, AKT2 forms a weakly voltage‐dependent channel. However, patch‐clamp studies on AKT2‐transformed *Arabidopsis*, tobacco and poplar mesophyll cells displayed characteristics of a Ca^2+^‐ and pH‐ sensitive, K^+^ inward rectifier with pronounced differences from those found in heterologous expression systems (Ivashikina *et al.*, 2005; Langer *et al.*, 2002; Latz *et al.*, 2007). Moreover, a Ca^2+^ sensor CBL4 modulates K^+^ channel activity by promoting a kinase interaction‐dependent translocation of the channel to the plasma membrane (Gajdanowicz *et al.*, 2011).

The plant high‐affinity transporter HvHAK1 was first identified in barley and has sequence similarity with fungal HAK transporters. Subsequently, the HAK1s from *Arabidopsis* and rice have been isolated and characterized. Functional expression of HvHAK1, AtHAK5 and OsHAK in yeast mutants revealed that these transporters are responsible for the high‐affinity K^+^ uptake (Banuelos *et al.*, 2002; Gierth *et al.*, 2005; Santa‐Maria *et al.*, 1997). Direct electrophysiological evidence for a K^+^/H^+^ symporter with a stoichiometry of 1K^+^/1H^+^ was obtained using protoplasts from *Arabidopsis* root cells (Maathuis and Sanders, 1994), which could mediate K^+^ influx at submillimolar external K^+^. Transcript expression of *HAKs* is enhanced by K^+^ starvation, paralleling the onset of the high‐affinity K^+^ uptake (Armengaud *et al.*, 2009; Chen *et al.*, 2015).

In plants, drought phytohormone, ABA, and downstream nitric oxide (NO) and hydrogen peroxide (H_2_O_2_) signalling molecules regulate many key physiological processes including drought stress response (Shi *et al.*, 2014). In *Arabidopsis*, K^+^ starvation impacts nitrate reductase activity, NO_3_^−^ uptake and nitrate transporters (NRTs) (Armengaud *et al.*, 2009) and disruption of nitrate reductases and NO production affects K^+^ homoeostasis and stomatal regulation (Chen *et al.*, 2016). High cellular H_2_O_2_ can generate more hydroxyl radicals, resulting in K^+^ loss and programmed cell death (Apel and Hirt, 2004). Moreover, our previous study revealed that the drought‐tolerant wild barley XZ5 has highly efficient in K^+^ uptake and translocation mechanisms, especially under drought (Feng *et al.*, 2016; He *et al.*, 2015; Wang *et al.*, 2018).

Therefore, we hypothesized that HvAKT2 and HvHAK1 improve barley performance under drought. In this study, we revealed the K^+^ selectivity of AKT2 and HAK1 is originated from strepotophyte algae and AKT2 and HAK1 are evolutionarily conserved for K^+^ uptake in land plants. We found that overexpressing HvAKT2 and HvHAK1 improves drought tolerance in barley through the regulation of leaf H^+^ homoeostasis and cellular signalling.

## Results

### K^+^ selectivity of AKT2 and HAK1 originated from strepotophyte algae is evolutionarily conserved in plants

**Figure 1:**
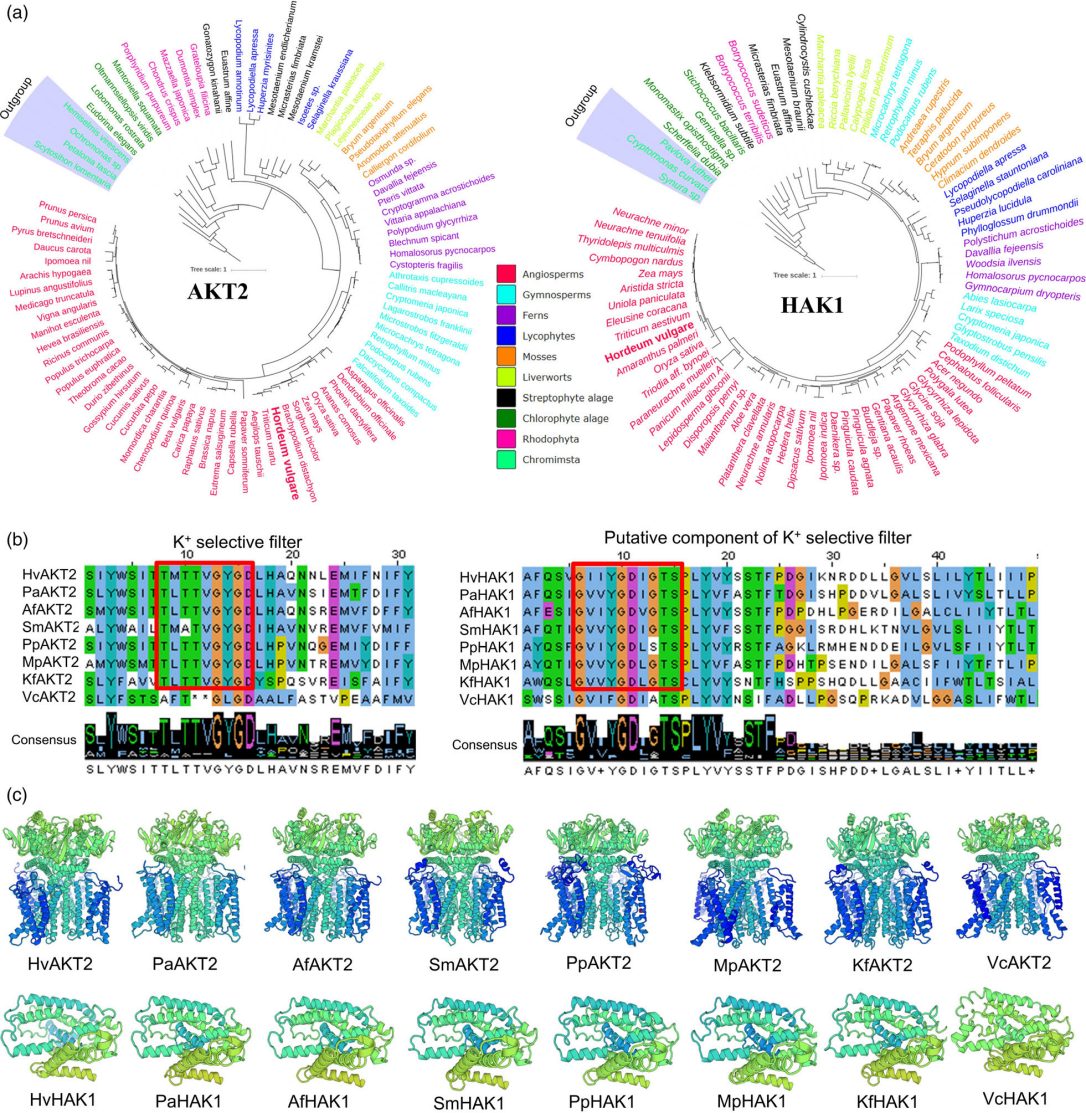
Phylogenetic trees, conserved domain alignment and predicted 3D structure of AKT2 and HAK1 proteins in plants and algae. (a) Phylogenetic trees of AKT2 and HAK1 proteins in representative species of major lineage of plants and algae (See Figure S1 for all 1KP species). The maximum likelihood was used to construct the trees. Clades are indicated by different colours. (b) Conserved domain alignment of AKT2s and HAK1s. The high conserved K^+^ selective filter in AKT2s and a putative K^+^ selective filter in HAK1s in eight representative plant and algal species. (c) Predicted 3D structure of AKT2s and HAK1s in eight representative plant and algal species. *Hv, Hordeum vulgare; Pa, Picea abies; Af, Azolla filiculoides; Sm, Selaginella moellendorffii; Pp, Physcomitrella patens; Mp, Marchantia polymorpha; Kf, Klebsormidium flaccidum; Vc, Volvox carteri*.

AKT2 and HAK1 form important parts of the plant low‐ and high‐affinity K^+^ uptake systems in plants. However, these genes have only been studied in limited number of angiosperm species. Here, we employed bioinformatics tools to explore the evolution of AKT2 and HAK1 in plant and algae (Figure 1a; Figure S1–S3; Table S1). Analysis using 1000 Plant Transcriptome database showed that the protein sequences of AKT2 and HAK1 are found in 80% and 75% of the 1,322 plant and algal species, respectively (Figure S1; Table S1). Among the major clades, AKT2 had a significant presence in Rhodophyta occurring in 57% of the tested species while only 2 out of 28 species of Rhodophyta contained HAK1. AKT2 and HAK1 were identified in 69% and 39% of the 117 chlorophyte algae species, respectively. Interestingly, AKT2 and HAK1 have evolved in 40 and 35 out of 54 streptophyte algae, respectively (Table S1), indicating a possible origin of the two genes of the high‐ and low‐affinity K^+^ uptake systems. Moreover, the hallmark of the K^+^ selective filter TxxTxGYGD in AKT2 and a putative K^+^ selective filter component GxxYGD was both found in significantly more species of streptophyte algae than in chlorophyte algae (Figure 1b; Table S1). Topology prediction, gene and protein evolution analysis, and evolutionary conservation analysis showed that HvAKT2 and HvHAK1 both have typical predicted K^+^ transport characteristics (Figures 1c, S2 and S3).

### HvAKT2 and HvHAK1 confer drought tolerance in barley

**Figure 2:**
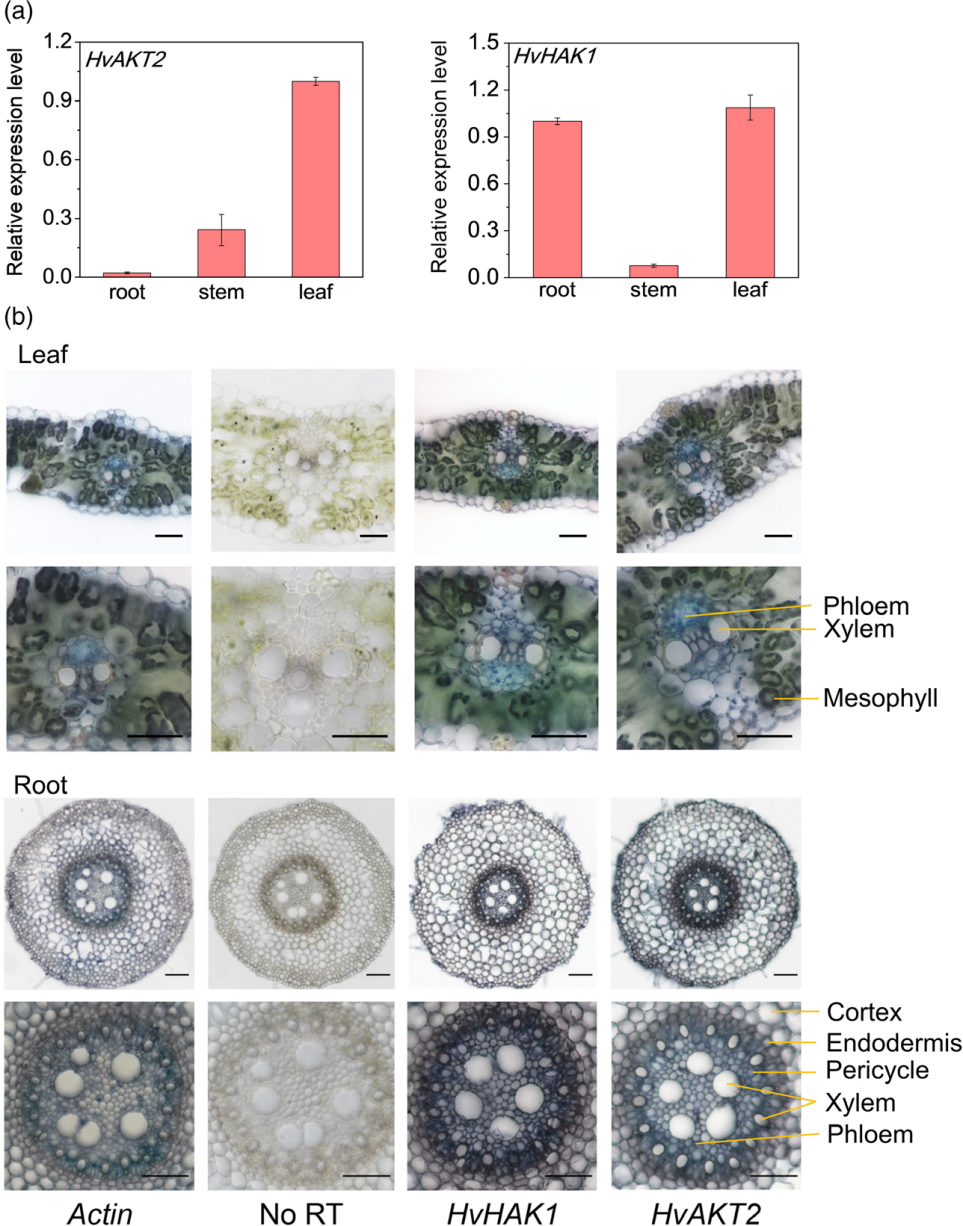
Tissue and cell‐type gene expression patterns of *HvAKT2* and *HvHAK1* in barley. (a) qRT‐PCR analysis of the relative transcript levels of *HvAKT2* and *HvHAK1* in different tissues of XZ5. (b) Tissue localization of *HvAKT2* and *HvHAK1* by in situ PCR. All samples were stained with BM Purple. The blue colour indicates the presence of digoxigenin (DIG)‐labelled cDNA. *HvActin* is a positive control. No reverse transcription (RT) is included as a negative control. Representative images are shown out of five replicates. Bars = 50 μm.

**Figure 3:**
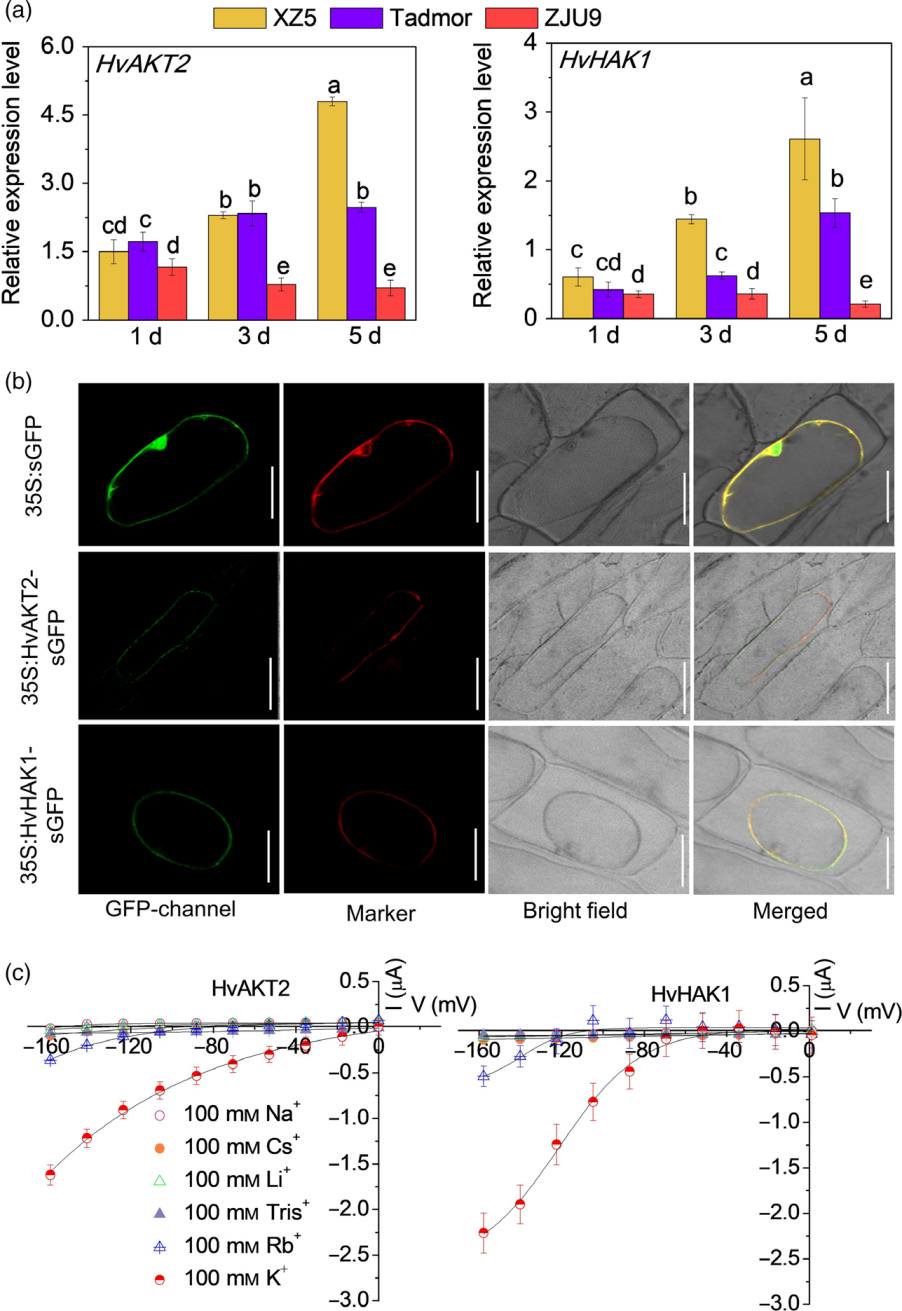
Subcellular localization and electrophysiology of HvAKT2 and HvHAK1. (a) Real‐time PCR analysis of *HvAKT2* and *HvHAK1* in three barley genotypes XZ5, Tadmor and ZJU9 subjected to PEG treatment. (b) Subcellular localization of the GFP, HvAKT2‐ and HvHAK1‐sGFP fusion proteins in onion epidermis cells. A plasma membrane RFP marker protein (pm‐rb CD3‐1008) was used as a reference. Bars = 50 μm. (c) Ion transport characteristics of HvAKT2 and HvHAK1 in *Xenopus laevis* oocytes. Holding potential was −20 mV, and voltage was clamped from −160 to 0 mV for 10 cycles. Data are mean ± SE (10–15 oocytes from at least three independent experiments).

**Figure 4:**
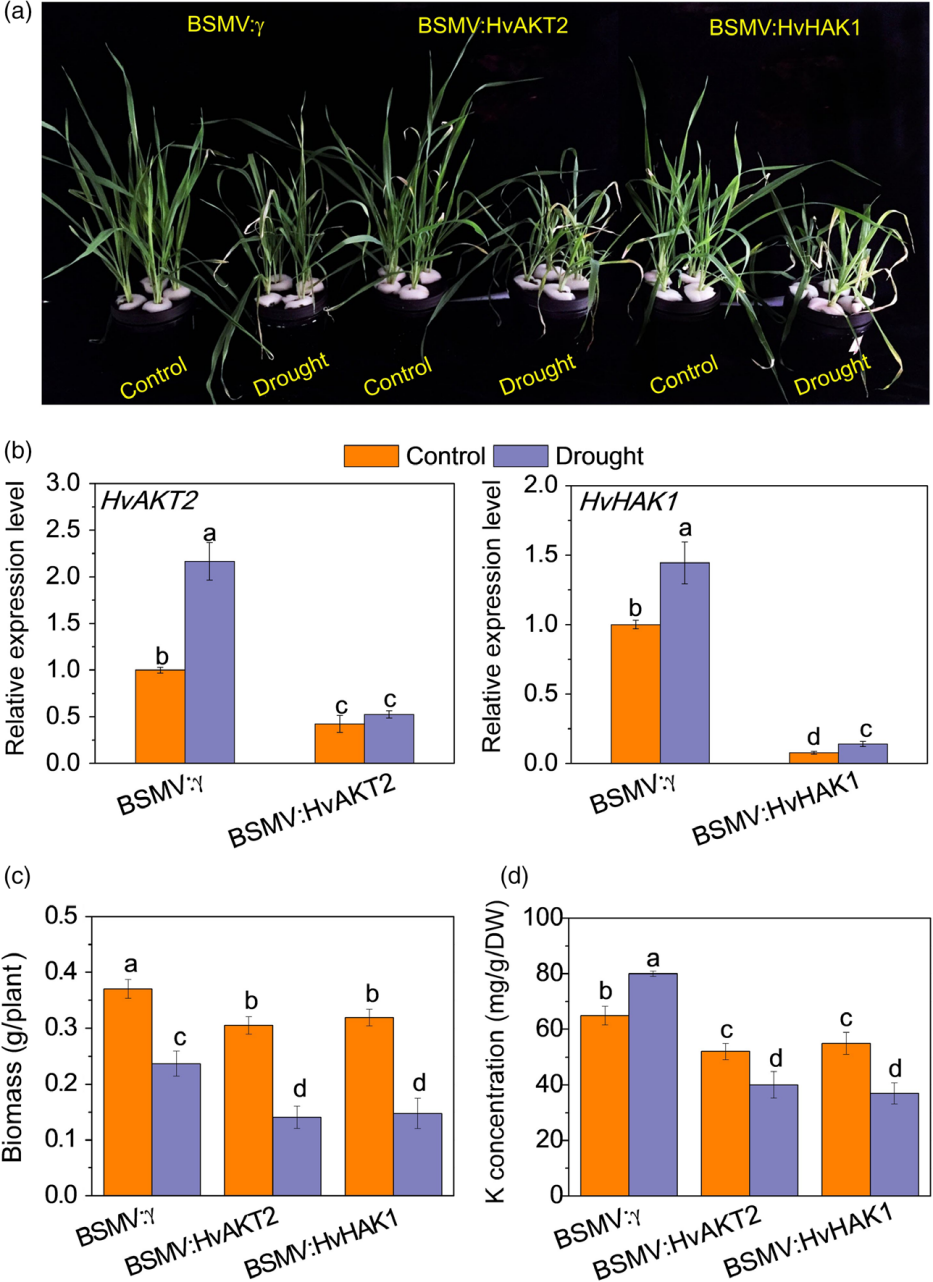
Functional assessment of *HvAKT2* and *HvHAK1 via* BSMV‐VIGS. (a) Phenotype after silencing of *HvAKT2* and *HvHAK1* in wild barley XZ5 *via* BSMV‐VIGS. Control and drought correspond to basic nutrition solution (BNS) and BNS + 20% PEG, respectively. (b) qRT‐PCR analysis of *HvHAK1* and *HvAKT2* in leaves of XZ5. (c) Biomass after silencing of *HvAKT2* and *HvHAK1* in XZ5 *via* BSMV‐VIGS. (d) K concentration in leaves of plants after silencing of *HvAKT2* and *HvHAK1* in XZ5 *via* BSMV‐VIGS. Inoculated seedlings were grown in BNS for 10 days, followed by PEG‐induced drought for 5 days. Data are mean ± SD (n = 6), and different letters indicate significant differences (*P < *0.05).

**Figure 5:**
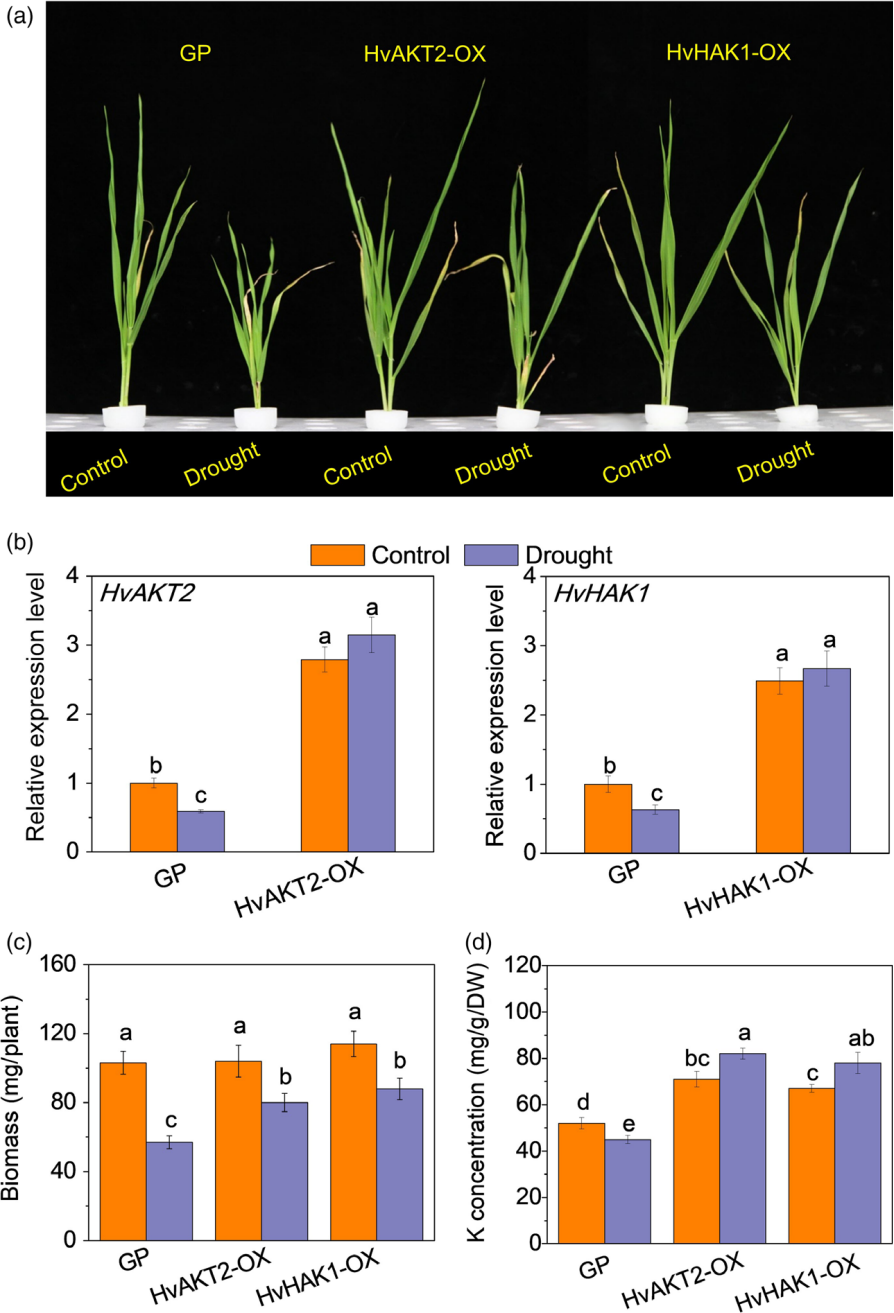
Functional assessment of *HvAKT2* and *HvHAK1 via* gene overexpression．(a) Phenotype in Golden Promise (GP) and overexpressed lines (OX). Control and drought correspond to basic nutrition solution (BNS) and BNS + 20% PEG (drought), respectively. (b) qRT‐PCR of *HvAKT2* and *HvHAK1* in two transgenic overexpression lines HvAKT2‐OX2 and HvHAK1‐OX2 (see Figure S6). (c) Biomass in GP and overexpressing barley lines. (d) K concentration in leaves of GP and overexpressing barley lines. Seedlings were grown in BNS for 10 days, followed by PEG‐induced drought for 5 days. Data are mean ± SD (n = 6), and different letters indicate significant differences (*P < *0.05).

We conducted a comprehensive set of experiments to functionally analyse HvAKT2 and HvHAK1 for their potential roles in barley drought tolerance (Figures 2, 3, 4, 5). Tissue‐specific gene expression in XZ5 showed that *HvAKT2* is mainly expressed in leaves, and *HvHAK1* is highly expressed in both roots and leaves, with slightly higher expression in leaves (Figure 2a). *In situ* PCR assay of *HvAKT2* and *HvHAK1* demonstrated that, in comparison with the positive (*HvACT*) and negative (No RT) controls (Figure 2b), the expression of *HvAKT2* is predominantly within the stele, particularly in vascular bundles—phloem and xylem of roots, but the expression of *HvHAK1* is in phloem and xylem cells, endodermis and epidermal cells of roots. In leaves, both *HvAKT2* and *HvHAK1* were mainly expressed in leaf mesophyll cells and vascular bundles particularly in phloem cells (Figure 2b). These results led us to focus on drought tolerance mechanism of leaf mesophyll tissue, where both *HvAKT2* and *HvHAK1* are highly expressed. Moreover, genotypic difference of *HvAKT2* and *HvHAK1* expression was also found between wild and cultivated barley accessions. Compared to the controls, 5‐d PEG treatment led to 4.8‐ and 2.6‐fold up‐regulation of *HvAKT2* and *HvHAK1* in the drought‐tolerant wild barley genotype XZ5, whereas *HvAKT2* and *HvHAK1* were significant down‐regulated in the drought‐sensitive cultivar ZJU9 (Figure 3a).

We then conducted cloning and expression of the *HvAKT2* and *HvHAK1* in onion epidermal cells and *Xenopus laevis* oocytes to analyse their subcellular localization and electrophysiological properties, respectively (Figures 3 and S4). GFP fluorescence of both HvAKT2 and HvHAK1 was overlapped with the plasma membrane RFP marker, indicating that HvAKT2 and HvHAK1 are localized in the plasma membrane (Figure 3b). Inward K^+^ currents were detected from *HvAKT2* and *HvHAK1* expressed oocytes in a [K^+^] dependent manner (Figure S4). HvAKT2 and HvHAK1 showed high selectivity for K^+^, low selectivity to Rb^+^ and no permeability to Na^+^, Cs^+^, Li^+^ and Tris^+^ (Figure 3c), validating the bioinformatics prediction (Figures 1, S1 and S3) that both HvAKT2 and HvHAK1 are highly K^+^ selective.

Disruption of *HvAKT2* and *HvHAK1* resulted in significant reduction of drought tolerance in XZ5 (Figure 4). The BSMV‐VIGS system was verified by inhibited expression of *HvPDS* by 95.3% in *PDS*‐inoculated XZ5 plants (Figure S5). Five days of PEG‐induced drought resulted in significantly greater leaf wilting and growth inhibition in BSMV:HvAKT2‐ and BSMV:HvHAK1‐inoculated plants compared to the mock‐inoculated ones (Figure 4a). The expression of *HvAKT2* and *HvHAK1* was suppressed in leaves of silenced plants by 68% and 93% under drought, respectively (Figure 4b), which was accompanied by significant decreases of biomass at 53.9% and 53.8% in drought treatment, respectively (Figure 4c). Drought treatment led to a significant increase of K^+^ concentration at 23.1% in the leaves of mock‐inoculated seedlings, while significant reductions at 23.2% and 32.7% were found in the leaves of BSMV:HvAKT2‐ and BSMV:HvHAK1‐inoculated plants, respectively (Figure 4d).

Overexpression of *HvAKT2* and *HvHAK1* caused a significant increase in drought tolerance in barley cultivar (wild type) Golden Promise (Figure 5). The gene expression in the transgenic lines HvAKT2‐OXs and HvHAK1‐OXs was validated with significant up‐regulation in four independent lines, and HvAKT2‐OX2 and HvHAK1‐OX2 were selected for further experiments (Figure S6). Drought‐induced large growth inhibition in the wild type was significantly mitigated in the HvAKT2‐OX and HvHAK1‐OX plants (Figure 5a). PEG‐induced drought treatment slightly increased the transcripts of already overexpressed of *HvAKT2* and *HvHAK1*, which were significantly decreased in Golden Promise (Figure 5b). In contrast to the control, drought decreased biomass of Golden Promise by 44.7%, but the biomass reduction was 23.1% and 22.8% in HvAKT2‐OX and HvHAK1‐OX plants, respectively (Figure 5c). Strikingly, gene overexpression led to a significant increase of leaf K^+^ concentration by 15.5% and 16.4% in the leaves of HvAKT2‐OX and HvHAK1‐OX plants subjected to drought, respectively, while it was decreased by 13.5% in Golden Promise (Figure 5d).

### Silencing and overexpressing of HvAKT2 and HvHAK1 modulate HvHA1 expression, H^+^‐ATPase activity and H^+^ flux in leaves

**Figure 6:**
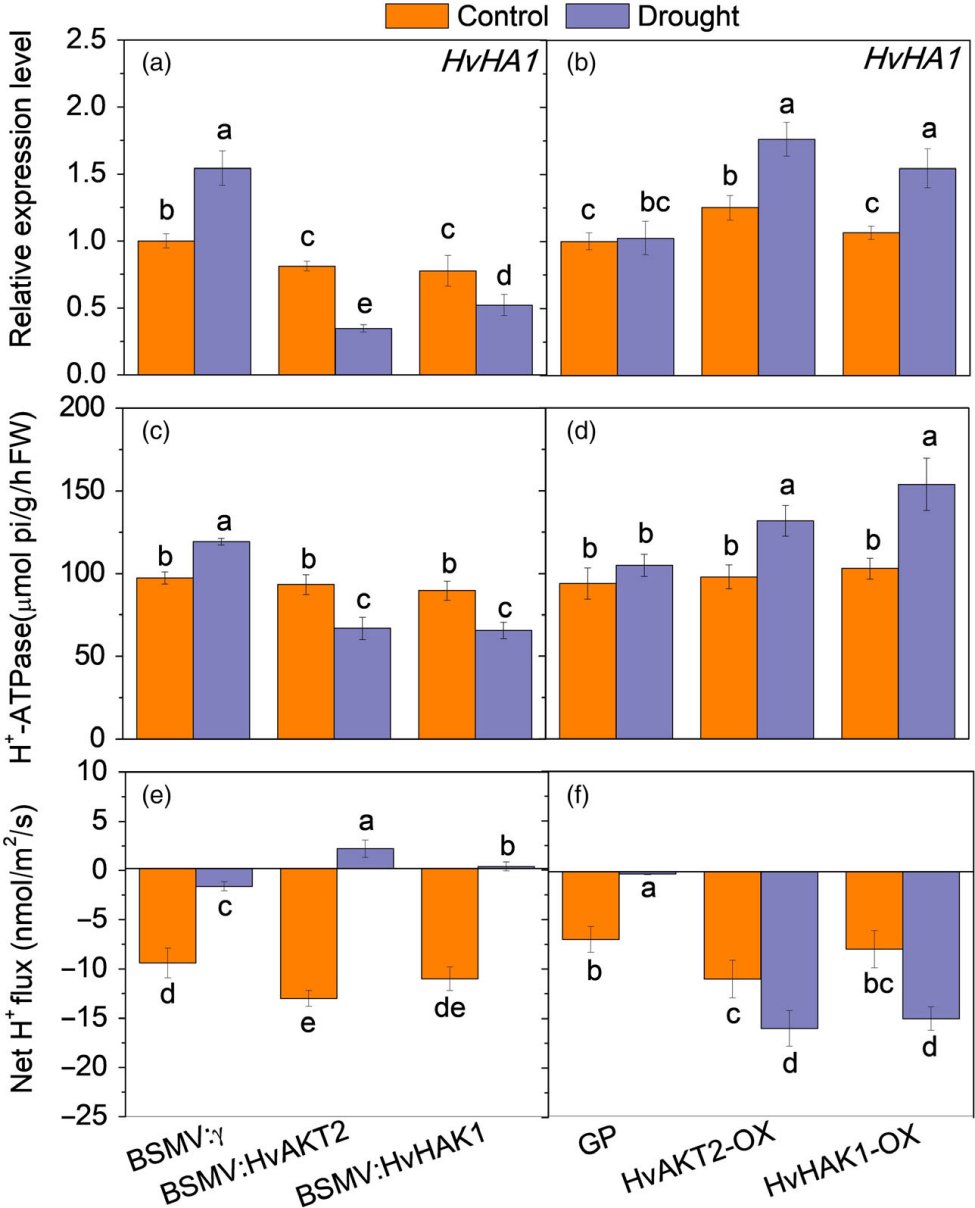
Drought‐induced expression of *HvHA1*, H^+^‐ATPase enzyme activity and H^+^ flux in leaves of transgenic barley lines. qRT‐PCR analysis of *HvHA1* (a, b), activity of H^+^‐ATPase (c, d) and H^+^ fluxes (e, f) in leaves of silenced and overexpressed plants in the control and drought. Seedlings were grown in BNS for 10 days, followed by PEG‐induced drought for 5 days. Steady‐state H^+^ fluxes were measured over 0h (control) and 1h (PEG‐induced drought). Data are mean ± SD (n = 6), and different letters indicate significant differences (*P < *0.05).

Five putative *HvHAs* genes including *HvHA1* encode the H^+^‐ATPases in barley in the wild barley genome (Dai *et al.*, 2018). Surprisingly, we found that, in response to drought, *HvHA1* expression in leaves was significantly down‐regulated by 57.4% and 32.9% in the BSMV:HvAKT2‐ and BSMV:HvHAK1‐inoculated plants, respectively (Figure 6a). On the contrary, drought significantly up‐regulated *HvHA1* by 40.8% and 45.2% in HvAKT2‐OX and HvHAK1‐OX plants, respectively, without affecting the *HvHA1* transcripts in leaves of Golden Promise (Figure 6b). Consistently, in response to drought, H^+^‐ATPase activity was significantly decreased by 31.2% and 32.6% in leaves of BSMV:HvAKT2‐ and BSMV:HvHAK1‐inoculated plants, respectively (Figure 6c). H^+^‐ATPase activity was increased in leaves of HvAKT2‐OX, and HvHAK1‐OX plants by 34.7% and 49.5% in response to drought, while there was a little change in Golden Promise (Figure 6d). Similar results were found in roots of these transgenic lines (Figure S8). Moreover, drought (1‐h in 20% PEG) led to a reverse of H^+^ fluxes from efflux (net H^+^ release) to influx (net H^+^ uptake) in leaf mesophyll of drought‐sensitive BSMV:HvAKT2‐ and BSMV:HvHAK1‐inoculated plants (Figure 6e). On the contrary, overexpression of *HvAKT2* and *HvHAK1* significantly increased the H^+^ efflux from leaf mesophyll in response to drought as compared to that in Golden Promise (Figure 6f).

### K^+^, H^+^ and Ca^2+^ fluxes in leaves silencing and overexpressing of HvAKT2 and HvHAK1 reveal coordinated ion homoeostasis for drought tolerance of barley

**Figure 7:**
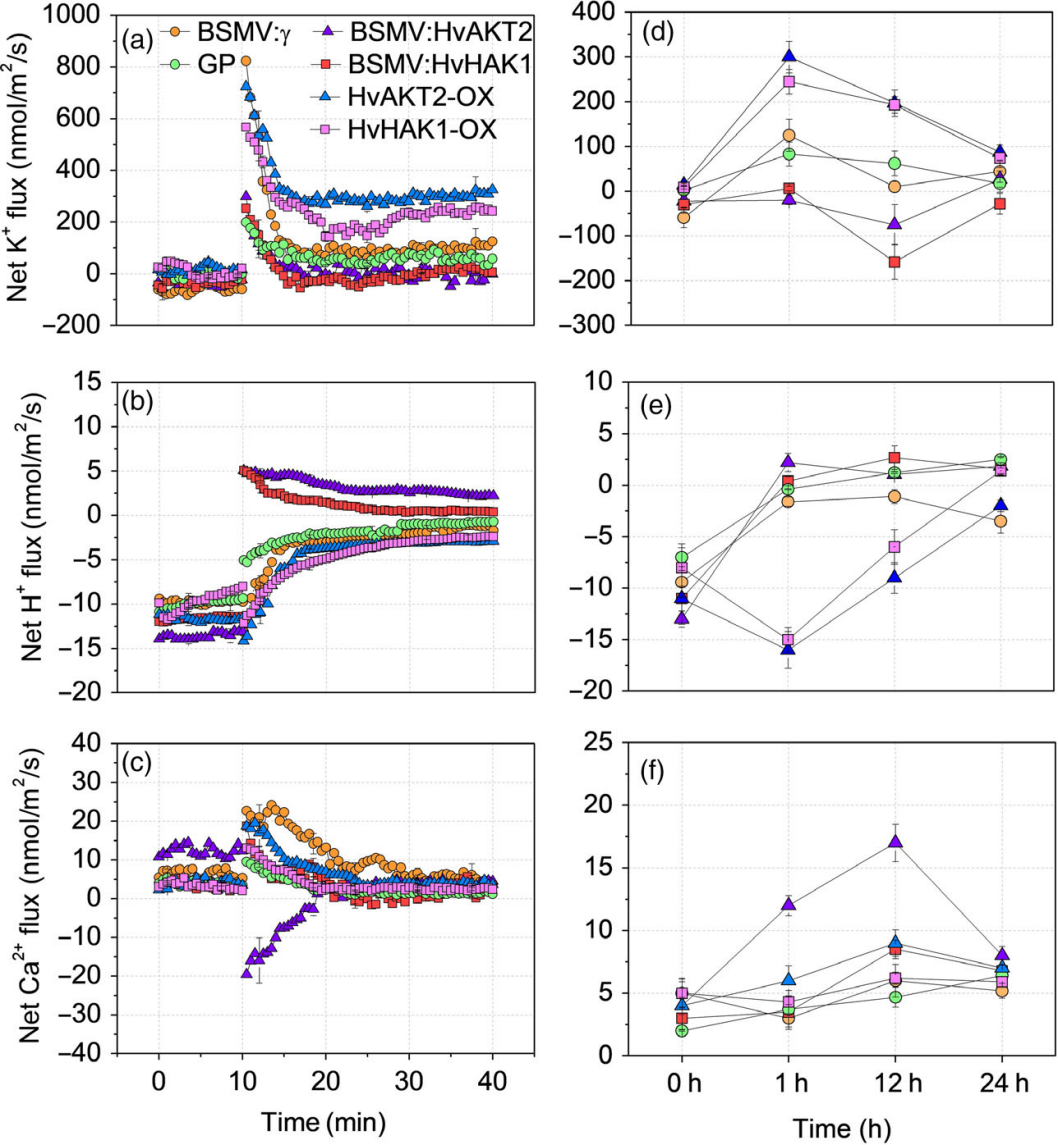
Drought‐induced ion fluxes in leaves of transgenic barley lines. Transient (a, b, c) and steady‐state (d, e, f) changes in K^+^, H^+^ and Ca^2+^ fluxes from leaf mesophyll of silenced and overexpressed plants subjected to 20% PEG‐induced drought treatment. Excised leaves were pre‐incubated in the BSM (0.5 mm KCl and 0.1 mm CaCl_2_) for 2 h. For transient ion fluxes, data are means ± SE (n = 8). Steady‐state ion fluxes were measured over 0, 1, 12 and 24 h after PEG exposure. Data are mean ± SE (n = 10).

We then conducted K^+^, H^+^ and Ca^2+^ flux measurements in all barley lines (XZ5, silencing lines, Golden Promise and overexpressing lines) and over 24‐h time course to decipher the roles of *HvAKT2* and *HvHAK1* in barley drought tolerance. In leaf mesophyll, silencing *HvAKT2* and *HvHAK1* caused significantly reduced K^+^ uptake after 1 and 12 h under PEG‐induced drought as compared to the control (Figure 7a,d). Interestingly, mock‐inoculated plants displayed H^+^ efflux in leaves of the controls and plant subjected to the PEG‐induced drought treatment after 1, 12 and 24 h, while *HvAKT2‐* and *HvHAK1‐*silenced plants showed a PEG‐induced H^+^ influx (Figure 7b,e). Leaf mesophyll cell Ca^2+^ fluxes in BSMV:HvHAK1 plants significantly increased after 1 and 12 h of drought (Figure 7c,f). Similar trends were observed for K^+^, H^+^ and Ca^2+^ fluxes in roots of the silenced lines (Figure S9).

In leaf mesophyll of plants overexpressing *HvAKT2* and *HvHAK1*, PEG‐induced drought stress resulted in 2.6‐ and 1.8‐fold higher K^+^ influx in HvAKT2‐OX and HvHAK1‐OX plants than that of Golden Promise (Figure 7a). The K^+^ influx decreased after 24 h of PEG treatment among all plants, but remained significantly higher K^+^ uptake (70‐80 nmol m^−2^ s^−1^) in the overexpressing lines than that of the control (Figure 7a,d). Interestingly, plants overexpressing *HvAKT2* and *HvHAK1* maintained significantly larger H^+^ efflux in the control and PEG‐induced drought treatment after 1 and 12 h than those of Golden Promise (Figure 7b,e). Ca^2+^ fluxes of leaf mesophyll cells in HvAKT2‐OX plants were significantly increased after 1 and 12 h of drought (Figure 7c,f).

### Leaf NO and H_2_O_2_ signalling is affected by silencing and Overexpressing HvAKT2 and HvHAK1

**Figure 8:**
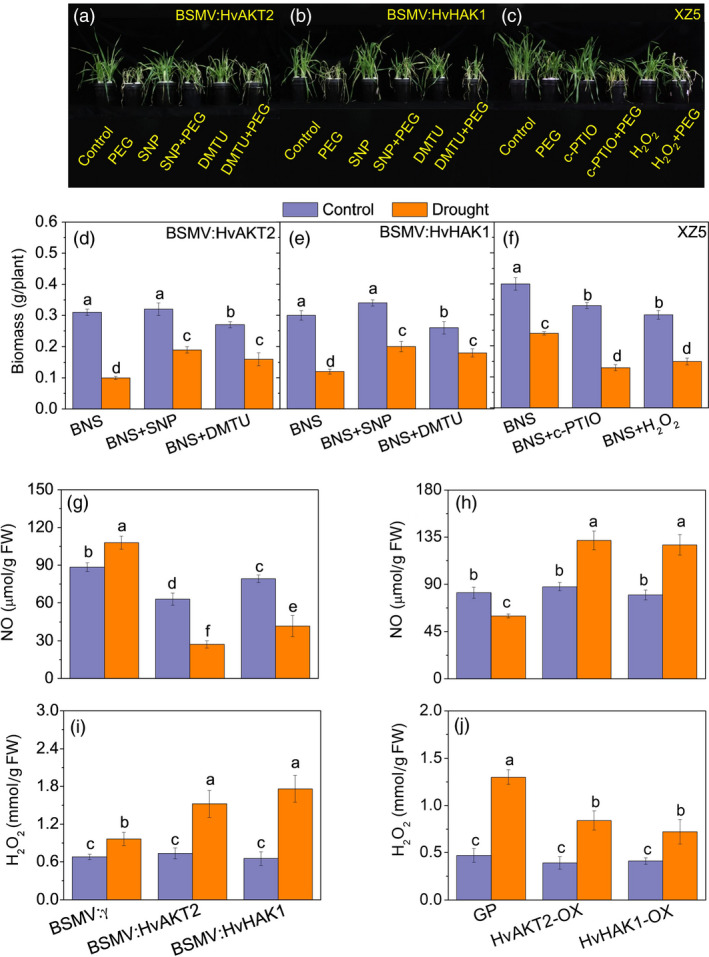
Role of NO and H_2_O_2_ in the drought response of transgenic barley lines. Phenotype (a, b) and biomass (d, e) of the wild barley, XZ5, subjected to drought stress after silencing of *HvAKT2* and *HvHAK1* after addition of exogenous SNP (NO donor) and DMTO (H_2_O_2_ scavenger) Phenotype (c) and biomass (f) of the wild barley, XZ5, subjected to drought stress after addition of c‐PTIO (NO scavenger) and exogenous H_2_O_2_. NO (g, h) and H_2_O_2_ (i, j) content in leaves of silenced and overexpressed plants. Seedlings were grown in BNS for 10 days, followed by PEG‐induced drought for 5 days. Data are mean ± SD (n = 6), and different letters indicate significant differences (*P < *0.05). Control, basic nutrition solution (BNS); Drought, BNS + 20% PEG‐induced drought.

NO and H_2_O_2_ are two key secondary messengers for drought response in plant cells (Chen *et al.*, 2016; Zhao *et al.*, 2019). Addition of the NO donor sodium nitroprusside (SNP) and H_2_O_2_ scavenger dimethyl thiourea (DMTU) significantly alleviated drought symptoms in *HvAKT2* and *HvHAK1* silencing plants (Figure 8a,b). Drought‐induced biomass reduction in plants with silenced *HvAKT2* and *HvHAK1* was 67.7% and 60% under PEG treatment, but the biomass decrease was significantly less at 40.6% and 41.2% with SNP and 40.7% and 30.8% with DMTU, respectively (Figure 8d,e). In contrast, exogenous NO scavenger c‐PTIO and H_2_O_2_ in addition to the PEG treatment decreased drought tolerance of XZ5 (Figure 8c), where significantly larger drought‐induced decrease of biomass in XZ5 was 60.1% and 50% after addition of c‐PTIO and H_2_O_2_ to PEG treatment, respectively (Figure 8f).

Leaf NO content decreased significantly in BSMV:HvAKT2‐ and BSMV:HvHAK1‐inoculated plants in response to PEG treatment, in contrast to a significant 22.3% increase of NO content in leaves of mock‐inoculated plants (Figure 7d). Drought significant significantly increased NO content in the leaves of HvAKT2‐OX, and HvHAK1‐OX transgenic plants as compared to that of Golden Promise (Figure 8h). Moreover, drought‐induced increase of leaf H_2_O_2_ contents was significantly larger in *HvAKT2‐* and *HvHAK1‐*silenced lines compared to the mock plants (Figure 8i). In contrast, drought treatment resulted in a highly significant increase in H_2_O_2_ content in Golden Promise, but a smaller increase was observed in the HvAKT2‐OX and HvHAK1‐OX lines (Figure 8j).

## Discussion

### AKT2 and HAK1 are evolutionarily conserved for potassium uptake and stress response in plants

Land plants have evolved to thrive in the terrestrial environment, where K^+^ availability is dependent on the soil type, rainfall and many environmental factors. High‐ and low‐affinity K^+^ uptake systems have enabled plants to adapt to the low K^+^ land lifestyle (Cao *et al.*, 1995; Rodriguez‐Navarro and Rubio, 2006; Wang and Wu, 2013). The evolution of high‐ and low‐affinity K^+^ uptake mechanisms was a prerequisite for the colonization of land by plants (Grabov, 2007). AKT2s and HAK1s have been found in evolutionarily diverse organisms ranging from green algae to angiosperms (Figure 1; Figure S1–S3; Table S1). The presence of AKT2s and HAK1s in plant genomes (Chen *et al.*, 2017) implies that they play an important role in K^+^ acquisition, allowing plants to survive in potassium‐poor environments. Importantly, our data showed that the presence and structure of AKT2s and HAK1s are highly conserved in green plants and are likely to originate from streptophyte algae (Figure 1; Figure S1–S3; Table S1). Thus, it might be expected that high‐affinity and low‐affinity K^+^ uptake systems that land plants possess for acclimation and adaptation to the variable and harsh terrestrial environments existed in their ancestors.

Plant inward‐rectifying K^+^ channels harbour regulatory domains comprising a putative cyclic nucleotide‐binding site, a KHA region and an ankyrin domain (Jan and Jan, 1997; Véry and Sentenac, 2003). A unique fingerprint of K^+^ channels is the highly conserved selectivity filter motif TxxTxGYGD, allowing the highly selective passage of K^+^ ions across the membrane (Doyle *et al.*, 1998; Jegla *et al.*, 2018; Riedelsberger *et al.*, 2015). Mutations in the selectivity filter can fundamentally alter the permeation properties of the channels (Dreyer and Uozumi, 2011). The KAT1‐like channel from melon with unusual K^+^⁄Na^+^ permeation/blocking properties (Zhang *et al.*, 2011) indicates that the channel pores have evolved to balance selectivity over a wide spectrum of potential competing ions. Given the evolutionary importance of plant K^+^ channels, changes in ion selectivity may affect the evolution of K^+^ transporters for plant adaptation to land. In our study, we found that the full TxxTxGYGD motif of AKT2s appeared in streptophyte algae but not in chlorophyte algae (Figure 1; Figure S1–S3; Table S1). Although there are many extant land‐dwelling chlorophyte algae, a group of freshwater streptophyte algae represents the lineage that is sister to embryophytes. Environmental stress tolerance features of streptophyte algae may have facilitated their transition to land (Becker and Marin, 2009; Zhao *et al.*, 2019). Meanwhile, the current study demonstrated that heterologous expression of *HvAKT2* and *HvHAK1* in *Xenopus laevis* oocytes shows higher selectivity to K^+^ over other cations (Figures 3d and S3). These data indicated that AKT2 and HAK1 have become conserved for potassium uptake and stress response in plants.

### Overexpressing HvAKT2/HvHAK1 enhances H^+^ homoeostasis for efficient K^+^ uptake under drought

Plant K^+^ uptake is mediated by low‐ and high‐affinity transport systems taking advantage of the electrical gradient and the proton motive force established by H^+^‐ATPase (Dreyer and Uozumi, 2011; Epstein *et al.*, 1963; Palmgren, 2001; Shabala *et al.*, 2016; Wang *et al.*, 2014). However, little evidence for the interaction of K^+^ transport and H^+^ pumping for drought tolerance has been obtained using transgenic plants. Here, we present solid experimental results that the expression of *HvHA1*, H^+^‐ATPase activity and H^+^ efflux is highly induced in the barley lines overexpressing *HvAKT2* and *HvHAK1* (Figure 6).

*AKT2,* which encodes a K^+^ channel subunit, shows gating similar to AKT1/KAT1 channels (Mode 1) and gating of little voltage sensitivity (Mode 2) (Dreyer *et al.*, 2001; Xicluna *et al.*, 2007). Switching between the two gating modes is under post‐translational control (Dreyer and Uozumi, 2011; Michard *et al.*, 2005). Interestingly, shifting AKT2 from Mode 1 to the voltage‐independent Mode 2 efficiently assists the plasma membrane H^+^‐ATPase in energizing transmembrane transport processes (Gajdanowicz *et al.*, 2011). Here, we showed that overexpression of *HvAKT2* in barley leaves increases *HvHA1* expression, enzyme activity of H^+^‐ATPase and high H^+^ efflux (Figures 6 and 7) in response to drought, leading to drought tolerance (Figure 5). It is also likely that increasing the expression of *HvAKT2* provides more chance for Ca^2+^ sensing and phosphorylation in barley plants, especially under stress conditions, similar to other types of ion channels (Chen *et al.*, 2010; Shabala *et al.*, 2019). Indeed, an *Arabidopsis* phosphatase of type 2C interacts with AKT2, which links the AKT2 phosphorylation status to the ABA signalling pathway and drought (Chérel *et al.*, 2002), and an *Arabidopsis* plasma membrane‐localized receptor‐like kinase, MRH1, post‐translationally regulates AKT2 (Sklodowski *et al.*, 2017). Plant ion channels are usually low abundant proteins due to their high transport capacity. Therefore, plants only need to express between 10 and 1,000 ion channels as compared to 10^5^‐10^6^ pumps per cell to control ion homoeostasis (Hills *et al.*, 2012; Latz *et al.*, 2007; Shabala *et al.*, 2019). Channels and pumps often co‐localize in a cell membrane, indicating potential interactions. For instance, the *Arabidopsis* slow anion channel, SLAC1, interacts with most of the Shaker channels present in guard cells, including AKT2 (Zhang *et al.*, 2016). Therefore, a physical interaction between HvAKT2 and HvHAs may occur given the overexpression of HvAKT2 and huge number of the HvHAs at plasma membrane in transgenic barley lines. This is the first report showing this potential interaction of AKT2 with HvHA1 to actively regulate plant drought tolerance. However, this physical interaction requires future investigation.

The association between HAK1 and HvHA1 is more pronounced because HAK1 is a high‐affinity K^+^ transporter that requires H^+^ for co‐transporting K^+^ (Grabov, 2007). HvHAK1 transporter from barley is likely to represent Epstein's high‐affinity uptake system (Epstein *et al.*, 1963; Santa‐Maria *et al.*, 1997). Expression of *HvHAK1* orthologues in tomato (*Lycopersicon esculentum*; *LeHAK5*) and *Arabidopsis* (*AtHAK5*) was activated by low external K^+^ (Ahn, 2004; Grabov, 2007). Moreover, transgenic *Arabidopsis* expressing barley *HvHAK1* showed enhanced K^+^ uptake under K^+^ deprivation (Fulgenzi *et al.*, 2008) and transgenic rice seedlings overexpressing *OsHAK1* exhibited higher tolerance to drought stress than control plants (Ahmad *et al.*, 2016; Chen *et al.*, 2015). Therefore, HvHAK1 is a key co‐transporter fuelled by H^+^‐ATPase to improve drought tolerance in barley (Figures 4, 5, 6, 7, 8).

### HvAKT2 and HvHAK1 modulate ion homoeostasis and signalling for drought tolerance in barley

An important response of plants to drought stress is the uptake of K^+^ (Shabala and Pottosin, 2014). Inward‐rectifying K^+^ channels such as AKT1 and AKT2 in plants provide major pathways for low‐affinity K^+^ uptake (Véry and Sentenac, 2003). In *Arabidopsis*, AKT2 is up‐regulated by salinity and ABA, suggesting that AKT2 is involved in the recirculation of K^+^ through the phloem (Shabala and Cuin, 2008) and in drought tolerance (Lacombe *et al.*, 2000). Also, AKT2 currents are reduced in the presence of the phosphatase AtPP2CA (Chérel *et al.*, 2002) and enhanced by CBL4/SOS3 and CIPK6 (Held *et al.*, 2011). Moreover, High‐affinity K^+^ uptake is coupled with the transport of H^+^ through the H^+^/K^+^ symporters (Banuelos *et al.*, 2002; Rodriguez‐Navarro and Rubio, 2006), but the molecular regulation of HAK1s in plant drought tolerance is less studied. Here, we show that drought stress causes a high K^+^ influx in plants overexpressing *HvAKT2* and *HvHAK1*, but it was significantly reduced in the gene‐silenced plants (Figure 7), which may be due to the activation of low‐ and high‐affinity K^+^ uptake systems.

Plant apoplastic pH is sensitive to soil moisture and drought stress, and the alteration of pH could be a chemical signal that affects ion transport (Bacon *et al.*, 1998). Low apoplastic pH dramatically down‐regulates AKT2 activity, and even small physiological cytosolic pH variations substantially affect the rectification properties of AKT2 (Lacombe *et al.*, 2000). Here, we demonstrated a dramatic H^+^ influx in leaf mesophyll of plants silencing *HvAKT2* and *HvHAK1* under drought stress, while control plants displayed less H^+^ influx (Figure 7b,e). It suggests that leaf apoplastic alkalization caused by reduced H^+^ efflux may be a key reason for drought sensitivity in the *HvAKT2‐* and *HvHAK1‐*silenced plants. However, further study is necessary to demonstrate whether this apoplastic alkalization is accompanied by membrane depolarization.

NO is a key cellular signal tightly associated with K^+^ nutrition (Armengaud *et al.*, 2009; Chen *et al.*, 2016), and NO deactivates guard cell inward K^+^ currents through a process that involves Ca^2+^ signalling (Garcia‐Mata *et al.*, 2003). Here, NO content decreased in plants silencing *HvAKT2* and *HvHAK1* and increased in plants overexpressing *HvAKT2* and *HvHAK1* under drought stress (Figure 8). Hydrogen peroxide (H_2_O_2_) is an important secondary messenger for plant stress signalling and regulation of K^+^ homoeostasis (Apel and Hirt, 2004; Wang *et al.*, 2017). H_2_O_2_ is an early response of K^+^ deficiency and externally added H_2_O_2_ was sufficient for the induction of the high‐affinity K^+^ uptake (Shin and Schachtman, 2004; Wang *et al.*, 2017). Although there was no report on the correlation between AKT2 and HAK1 regulated K^+^ homoeostasis and H_2_O_2_ accumulation in plant drought tolerance, positive relationships between H_2_O_2_ production and K^+^ accumulation under salt stress were found for *Arabidopsis*, cucumber (*Cucumis sativus*) and pumpkin (*Cucurbita moschata*) (Huang *et al.*, 2019; Ma *et al.*, 2012; Redwan *et al.*, 2016). For instance, it was demonstrated that higher salt tolerance in pumpkin is related to its higher K^+^ uptake and H_2_O_2_ accumulation in the root apex. This was associated with salt‐induced higher expression of key differentially expressed genes (DEGs) such as *respiratory burst oxidase homolog D* (*RBOHD*), *14‐3‐3 protein* (*GRF12*), *plasma membrane H^+^‐ATPase* (*AHA1*) and *HAK5* in pumpkin than those in cucumber. Knocking out of RBOHD in pumpkin via genome editing led to a salt‐sensitive phenotype: lower root apex H_2_O_2_ and K^+^ content and *GRF12*, *AHA1* and *HAK5* expression. Therefore, salt tolerance in pumpkin is regulated by RBOHD‐dependent transcriptional and post‐translational activation of AHAs operating upstream of HAK5 (Huang *et al.*, 2019). In this study, H_2_O_2_ content increased significantly in plants silencing *HvAKT2* and *HvHAK1*, but significantly reduced in overexpression plants under drought stress, which were also linked to altered K^+^ and H^+^ homoeostasis and drought tolerance in barley (Figures 4, 5, 6, 7, 8). In summary, these results indicated that NO and H_2_O_2_ may regulate low‐ and high‐affinity K^+^ uptake systems through enhanced H^+^ homoeostasis in *HvAKT2* and *HvHAK1* overexpressing barley lines to enhance drought tolerance.

## Conclusions

In this study, significant correlations between the key traits (Table S6) indicated that highly coordinated K^+^ transport and accumulation, H^+^ flux and H^+^‐ATPase activity, gene expression, and NO and H_2_O_2_ signalling are key mechanisms for drought tolerance of barley overexpressing *HvAKT2* and *HvHAK1*. Therefore, we propose that HvAKT2 and HvHAK1 contribute to drought stress tolerance in barley, implying that both high and low‐affinity K^+^ uptake mechanisms play essential roles in plant drought tolerance. Future research should focus on the manipulation of both K^+^ uptake systems using genome editing technology to not only improve the nutrient use efficiency for sustainable agriculture but also enhance drought tolerance of crops to better adapt in a changing climate.

## Materials and methods

### Plant materials

A range of barley lines were used in this study including the drought‐tolerant Tibetan wild barley genotype XZ5 (*Hordeum vulgare* L. ssp. *spontaneum*), the drought‐tolerant barley (*Hordeum vulgare* L.) cultivar Tadmor, the drought‐sensitive cultivar ZJU9, the wild‐type Golden promise for barley transformation, and barley stripe mosaic virus‐induced gene silencing (BSMV‐VIGS) and overexpression lines.

### Evolutionary bioinformatics

Evolutionary bioinformatics were conducted as described in Zhao *et al.,* (2019). Briefly, candidate protein sequences of AKT2s and HAK1s were selected using the 1000 Plant Transcriptome (1KP) (Leebens‐Mack *et al.*, 2019) database that satisfied the criteria of E‐value and query coverage at different levels (www.onekp.com). Phylogenies were constructed with FastTree using the maximum likelihood, and the Interactive Tree of Life resource (https://itol.embl.de/) was used to annotate phylogenetic trees. Protein and gene evolutionary analysis of AKT2s and HAK1s in plant and algal species was conducted using PIECE (http://www.bioinfogenome.net/piece/) with inbuilt GLOOME and Exalign function. Protein sequence alignment was performed with Jalview. Functional domain was predicted by SMART (http://smart.embl-heidelberg.de/). 3D structure was predicted by SWISS‐MODEL (https://swissmodel.expasy.org/).

### Quantitative real‐time PCR

Quantitative real‐time PCR (qRT‐PCR) assays were conducted as described by Chen *et al.* (2016). Seven‐day‐old uniform seedlings were exposed to 1, 3 and 5 days of 20% PEG, and leaves were sampled from 2‐leaf‐stage plants after drought treatment. Total RNA was isolated with the TaKaRa MiniBEST Plant RNA Extraction Kit, and first‐strand cDNA synthesis was carried out using the PrimeScipt^TM^ RT reagent Kit (Takara, Otsu, Japan). qRT‐PCR was performed on a CFX96 system machine using the SYBR Green Supermix (Bio‐Rad, Hercules, CA). The fold changes were expressed as 2^−ΔΔCt^ relative to the control. *HvACT* was used as a reference gene. Three biological and two technical replicates were conducted. Primers are listed in Table S2.

### In situ PCR

The *in situ* PCR analysis of *HvAKT2* and *HvHAK1* was performed as described by Ye *et al.* (2019) and Zhao *et al.* (2019). Briefly, barley leaves and roots were immersed in ice‐cold buffer. The samples were then embedded in 5% (w/v) agarose and sectioned to 60 μm (leaves) or 50 μm (roots). Ten sections were collected in a 200 µL tube to perform the DNase treatment, and RT‐qPCR was performed. Samples were then washed, incubated in alkaline phosphatase‐conjugated anti‐digoxigenin Fab fragments and developed in the dark with BM Purple AP substrate (Roche, Penzberg, Germany). The images were then taken with a Leica microscope. The positive control was carried out with *HvACT*, and no RT was used as a negative control. Primers are listed in Table S3.

### Molecular biology

Subcellular localization of expressed proteins was as described in Chen *et al.* (2011). The coding regions of *HvAKT2* and *HvHAK1* were amplified and cloned into pCAMBIA1300 with a CaMV 35S promoter:green fluorescent protein (35S:GFP) cassette to create fusion proteins. The final *35S:HvAKT2‐GFP* and *35S:HvHAK1‐GFP* fusion constructs were transiently expressed in onion epidermal cells using microprojectile bombardment. After 13–18 h, GFP was imaged after cell plasmolysis in 30% sucrose solution using confocal microscopy (Carl Zeiss Meditec AG, Jena, Germany). A plasma membrane RFP marker (pm‐rb CD3‐1008) was also used.

Barley stripe mosaic virus (BSMV) virus‐induced gene silencing (VIGS) was used according to He *et al.* (2015). The cDNA fragments of *HvPDS*, *HvAKT2 and HvHAK1* were amplified using primers containing *Nhe*I sites and inserted reversely into the RNAγ of BSMV to create cDNA clones of *BSMV:HvPDS*, *BSMV:HvAKT2* and *BSMV:HvHAK1* (primers are in Table S4). Sterilized seeds of XZ5 were germinated in an incubator (22/18 °C, day/night) for 7 days, and then, uniform seedlings were transplanted to 1‐L containers filled with a modified Hoagland's solution (mg/L): KNO_3_, 101; Ca(NO_3_)_2_·4H_2_O, 236; MgSO_4_·7H_2_O, 98.4; NH_4_H_2_PO_4_, 23; HBO_3_, 0.185; MnCl_2_·4H_2_O, 0.099; NH_4_Mo_7_O_24_·4H_2_O, 1.236; ZnSO_4_·7H_2_O,0.115; CuSO_4_·5H_2_O, 0.05; Fe‐EDTA, 8.42. After inoculation, the seedlings were sprayed with diethyl pyrocarbonate (DEPC)‐treated water and covered with a transparent plastic to keep the high humidity for 3 days. *HvAKT2‐* and *HvHAK1‐*silenced seedlings were subjected to mock‐inoculation with BSMV:γ, BSMV:γ+20% PEG, BSMV:gene‐inoculated, and BSMV:gene+20% PEG treatments for 5 days. Six replicates were conducted.

Barley transformation was described in Bartlett *et al.* (2008). Open reading frames of *HvAKT2* and *HvHAK1* were PCR amplified, cloned into pDONR–Zeo (Invitrogen). Using Gateway technology (Invitrogen,Carlsbad, CA), the genes in the pDONR–Zeo vector were mobilized into the binary vector pBract214, which contains the *Ubiquitin* promoter. Subsequently, pBract214 and pSoup were introduced into the Agrobacterium strain AGL1 and infect embryo callus of Golden Promise. Regenerated seedlings were obtained 12 weeks after transformation. Evaluation of *HvAKT2‐OX* and *HvHAK1‐OX* transgenic barley lines was determined by real‐time PCR. Sterilized T_3_ seeds of the transgenic lines were germinated in an incubator (22/18 °C, day/night) for 7 days, and then, uniform seedlings were transplanted to 1‐L containers filled with half‐strength Hoagland's solution. The transgenic plants were grown in BNS for 10 days, and then, plants were subjected to BNS containing 20% PEG to examine the phenotype.

### Electrophysiology

Oocyte voltage clamp experiments were conducted according to Grefen *et al.* (2010) and Pornsiriwong *et al.* (2017). Briefly, *HvAKT2* and *HvHAK1* were cloned (see Table S5 for primers) using Gateway technology (Invitrogen,). pGEMHE‐DEST containing the ORF of *HvAKT2* and *HvHAK1* was linearized using *SbfI*‐HF (New England Biolabs, Ipswich, MA). The cRNA was synthesized using the mMESSAGE mMACHINET7 Kit (Ambion, Austin, TX), and cRNA (46 nL, 500 ng/µL) was injected into *Xenopus laevis* oocytes with a Nanoject II microinjector (Drummond Scientific, Broomall, PA). Oocytes were incubated in ND96 with gentamycin (5 mg/L), and currents were measured in HMg solution.

Net K^+^, H^+^ and Ca^2+^ fluxes were measured from leaf mesophyll cells using the noninvasive ion‐selective microelectrode (MIFE) technique (Feng *et al.*, 2016; Newman, 2001). For transient ion flux measurements, excised leaf and root segments were promptly immersed in a chamber for 2 h before measurements (Zeng *et al.*, 2014). Transient ion fluxes were measured for 10 min to ensure a steady flux, and then, 20% PEG were added for another 30 min. For steady‐state ion flux measurements, excised leaf and root segments were pretreated with 20% PEG for 0, 1, 12 and 24 h. Net ion fluxes were measured for 10–15 min. Net ion fluxes were measured from at least eight biological replicates.

### K measurement

The barley leaves and roots from hydroponic experiments were harvested and rinsed with deionized water. The samples were then dried at 80 °C for 72 h to a constant weight and were digested with HNO_3_ (Wu *et al.*, 2014). The levels of K were measured with an inductive‐coupled plasma (ICP)‐emission spectrophotometer (Optima 2100DV; PerkinElmer, Wellesley, MA).

### H^+^‐ATPase activity

H^+^‐ATPase activity was measured essentially as described by Regenberg *et al.* (1995) using an enzyme assay (Jiancheng Bio Co., Nanjing, China). The assay medium included 3 mm ATP and 0.02% Brij‐58. The barley leaf and root samples were pre‐incubated for 10 min with 0.02% Brij‐58. The reaction was initiated by the addition of 2 mg of samples to the assay medium.

### Measurement of NO and H_2_O_2_

Fresh leaves (0.3 g) were homogenized in 8 mL of 50 mm phosphate‐buffered saline (PBS) at pH 7.8 using a pre‐chilled mortar and pestle. Then, the homogenates were centrifuged at 10 000 ***g*** for 15 min and the supernatants were used for NO and H_2_O_2_ measurement. The NO content was measured with the nitrate reductase method using an NO assay kit, and the H_2_O_2_ content was determined with a H_2_O_2_ assay kit (Jiancheng Bio Co., Nanjing, China). For the experiments with the treatments of NO and H_2_O_2_, 200 µm NO scavenger 2‐(4‐Carboxyphenyl)‐4,4,5,5‐tetramethylimidazoline‐1‐oxyl‐3‐oxide (c‐PTIO), 200 µm NO donor SNP, 5 mm H_2_O_2_ scavenger N,N‐dimethylthiourea (DMTU) and 5 mm H_2_O_2_ were dissolved in distilled water and added to the nutrient solutions. Then, seedlings were subjected to 20% PEG treatments for 5 days in the hydroponic experiments.

### Statistical analysis

Statistical analyses were performed with a Processing System statistical software package using ANOVA followed by the Duncan's multiple range test.

## Conflict of interest

The authors declare no conflict of interest.

## Author contributions

FW, ZHC and XF planned and designed the research. XF, WL, CWQ, FZ and YW performed the experiments. XF, WL, ZHC and FW analysed the data. ZHC, FW, XF and GZ wrote the manuscript with contribution from all authors.

## Supporting information


**Figure S1** Phylogenetic analysis of AKT2 and HAK1 in different plant and algal species selected from the 1KP database.
**Figure S2** Protein evolutionary analysis of AKT2 and HAK1 using PIECE with an inbuilt GLOOME and an inbuilt Exalign function in plant and algal species.
**Figure S3** Functional domain of HvAKT2 and HvHAK1.
**Figure S4** Electrophysiology of HvAKT2 and HvHAK1 in *Xenopus laevis* oocytes.
**Figure S5** BSMV‐VIGS used in wild barley XZ5.
**Figure S6** Analysis of the expression of *HvAKT2* and *HvHAK1* in transgenic overexpression lines using qRT‐PCR.
**Figure S7** The K^+^ concentration in roots of silenced and overexpression lines.
**Figure S8** The activity of H^+^‐ATPase in roots of silenced and overexpression lines.
**Figure S9** Transient and steady‐state changes in K^+^, Ca^2+^ and H^+^ ﬂuxes from root epidermal cells of inoculated plants *via* BSMV‐VIGS subjected to 20% PEG treatments.
**Table S1** Statistics of evolution of AKT2s and HAK1s in the 1KP dataset
**Table S2** List of quantitative real‐time PCR primers
**Table S3** List of *in situ* PCR primers
**Table S4** List of primers for vector construction of BSMV:HvPDS, BSMV:HvAKT2 and BSMV:HvHAK1
**Table S5** List of primers for heterologous expression in *Xenopus laevis* oocytes and barley transformation
**Table S6** Correlation analysis among all parameters in different barley lines in the control and drought

## References

[pbi13332-bib-0001] Ahmad, I. , Devonshire, J. , Mohamed, R. , Schultze, M. and Maathuis, F.J. (2016) Overexpression of the potassium channel TPKb in small vacuoles confers osmotic and drought tolerance to rice. New Phytol. 209, 1040–1048. 26474307 10.1111/nph.13708

[pbi13332-bib-0002] Ahn, S.J. (2004) Expression of KT/KUP genes in *Arabidopsis* and the role of root hairs in K^+^ uptake. Plant Physiol. 134, 1135–1145. 14988478 10.1104/pp.103.034660 PMC389937

[pbi13332-bib-0003] Apel, K. and Hirt, H. (2004) Reactive oxygen species: Metabolism, oxidative stress, and signal transduction. Annu. Rev. Plant Biol. 55, 373–399. 15377225 10.1146/annurev.arplant.55.031903.141701

[pbi13332-bib-0004] Armengaud, P. , Sulpice, R. , Miller, A.J. , Stitt, M. , Amtmann, A. and Gibon, Y. (2009) Multilevel analysis of primary metabolism provides new insights into the role of potassium nutrition for glycolysis and nitrogen assimilation in *Arabidopsis* roots. Plant Physiol. 150, 772–785. 19346439 10.1104/pp.108.133629 PMC2689955

[pbi13332-bib-0005] Bacon, M.A. , Wilkinson, S. and Davies, W.J. (1998) pH‐regulated leaf cell expansion in droughted plants is abscisic acid dependent. Plant Physiol. 118, 1507–1515. 9847127 10.1104/pp.118.4.1507 PMC34769

[pbi13332-bib-0006] Banuelos, M.A. , Garciadeblas, B. , Cubero, B. and Rodriguez‐Navarro, A. (2002) Inventory and functional characterization of the HAK potassium transporters of rice. Plant Physiol. 130, 784–795. 12376644 10.1104/pp.007781 PMC166606

[pbi13332-bib-0007] Bartlett, J.G. , Alves, S.C. , Smedley, M. , Snape, J.W. and Harwood, W.A. (2008) High‐throughput Agrobacterium‐mediated barley transformation. Plant Methods, 4, 22. 18822125 10.1186/1746-4811-4-22 PMC2562381

[pbi13332-bib-0008] Becker, B. and Marin, B. (2009) Streptophyte algae and the origin of embryophytes. Ann. Bot. 103, 999–1004. 19273476 10.1093/aob/mcp044 PMC2707909

[pbi13332-bib-0009] Boscari, A. , Clement, M. , Volkov, V. , Golldack, D. , Hybiak, J. , Miller, A.J. , Amtmann, A. *et al.* (2009) Potassium channels in barley: cloning, functional characterization and expression analyses in relation to leaf growth and development. Plant Cell Environ. 32, 1761–1777. 19682291 10.1111/j.1365-3040.2009.02033.x

[pbi13332-bib-0081] Cai, S. , Chen, G. , Wang, Y. , Huang, Y. , Marchant, D.B. , Wang, Y. , Yang, Q. *et al.* (2017) Evolutionary Conservation of ABA Signaling for Stomatal Closure. Plant Physiol. 174, 732–747. 28232585 10.1104/pp.16.01848 PMC5462018

[pbi13332-bib-0010] Cao, Y.W. , Ward, J.M. , Kelly, W.B. , Ichida, A.M. , Gaber, R.F. , Anderson, J.A. , Uozumi, N. *et al.* (1995) Multiple genes, tissue‐specificity, and expression‐dependent modulation contribute to the functional diversity of potassium channels in *Arabidopsis* . Plant Physiol. 109, 1093–1106. 8552711 10.1104/pp.109.3.1093 PMC161413

[pbi13332-bib-0011] Chen, Z.H. , Hills, A. , Lim, C.K. and Blatt, M.R. (2010) Dynamic regulation of guard cell anion channels by cytosolic free Ca^2+^ concentration and protein phosphorylation. Plant J. 61, 816–825. 20015065 10.1111/j.1365-313X.2009.04108.x

[pbi13332-bib-0012] Chen, Z. , Grefen, C. , Donald, N. , Hills, A. and Blatt, M.R. (2011) A bicistronic, Ubiquitin‐10 promoter‐based vector cassette for transient transformation and functional analysis of membrane transport demonstrates the utility of quantitative voltage clamp studies on intact *Arabidopsis* root epidermis. Plant Cell Environ. 34, 554–564. 21251017 10.1111/j.1365-3040.2010.02262.x

[pbi13332-bib-0013] Chen, G. , Hu, Q. , Luo, L. , Yang, T. , Zhang, S. , Hu, Y. , Yu, L. *et al.* (2015) Rice potassium transporter OsHAK1 is essential for maintaining potassium‐mediated growth and functions in salt tolerance over low and high potassium concentration ranges. Plant Cell Environ. 38, 2747–2765. 26046301 10.1111/pce.12585

[pbi13332-bib-0014] Chen, Z.H. , Wang, Y. , Wang, J. , Babla, M. , Zhao, C. , Garcia‐Mata, C. , Sani, E. *et al.* (2016) Nitrate reductase mutation alters potassium nutrition as well as nitric oxide‐mediated control of guard cell ion channels in *Arabidopsis* . New Phytol. 209, 1456–1469. 26508536 10.1111/nph.13714

[pbi13332-bib-0015] Chen, Z.H. , Chen, G. , Dai, F. , Wang, Y. , Hills, A. , Ruan, Y.L. , Zhang, G. *et al.* (2017) Molecular evolution of grass stomata. Trends Plant Sci. 22, 124–39. 27776931 10.1016/j.tplants.2016.09.005

[pbi13332-bib-0016] Chérel, I. , Michard, E. , Platet, N. , Mouline, K. , Alcon, C. , Sentenac, H. and Thibaud, J. (2002) Physical and functional interaction of the *Arabidopsis* K^+^ channel AKT2 and phosphatase AtPP2CA. Plant Cell, 14, 1133–1146. 12034902 10.1105/tpc.000943 PMC150612

[pbi13332-bib-0017] Chérel, I. , Lefoulon, C. , Boeglin, M. and Sentenac, H. (2014) Molecular mechanisms involved in plant adaptation to low K^+^ availability. J. Exp. Bot. 65, 833–848. 24293613 10.1093/jxb/ert402

[pbi13332-bib-0018] Dai, F. , Wang, X. , Zhang, X.Q. , Chen, Z. , Nevo, E. , Jin, G. , Wu, D. *et al.* (2018) Assembly and analysis of a qingke reference genome demonstrate its close genetic relation to modern cultivated barley. Plant Biotechnol. J. 16, 760–770. 28871634 10.1111/pbi.12826 PMC5814578

[pbi13332-bib-0019] Doyle, D.A. , Morais, C.J. , Pfuetzner, R.A. , Kuo, A. , Gulbis, J.M. , Cohen, S.L. , Chait, B.T. *et al.* (1998) The structure of the potassium channel: molecular basis of K^+^ conduction and selectivity. Science, 280, 69–77. 9525859 10.1126/science.280.5360.69

[pbi13332-bib-0020] Dreyer, I. and Uozumi, N. (2011) Potassium channels in plant cells. FEBS J. 278, 4293–4303. 21955642 10.1111/j.1742-4658.2011.08371.x

[pbi13332-bib-0021] Dreyer, I. , Michard, E. , Lacombe, B. and Thibaud, J.B. (2001) A plant Shaker‐like K^+^ channel switches between two distinct gating modes resulting in either inward‐rectifying or ‘leak’ current. FEBS Lett. 505, 233–239. 11566182 10.1016/s0014-5793(01)02832-0

[pbi13332-bib-0022] Epstein, E. , Rains, D.W. and Elzam, O.E. (1963) Resolution of dual mechanisms of potassium absorption by barley roots. Proc. Natl Acad. Sci. USA, 49, 684. 16591089 10.1073/pnas.49.5.684 PMC299954

[pbi13332-bib-0023] Feng, X. , Liu, W. , Zeng, F. , Chen, Z. , Zhang, G. and Wu, F. (2016) K^+^ uptake, H^+^‐ATPase pumping activity and Ca^2+^ efflux mechanism are involved in drought tolerance of barley. Environ. Exp. Bot. 129, 57–66.

[pbi13332-bib-0024] Fulgenzi, F.R. , Peralta, M.L. , Mangano, S. , Danna, C.H. , Vallejo, A.J. , Puigdomenech, P. and Santa‐María, G.E. (2008) The ionic environment controls the contribution of the barley HvHAK1 transporter to potassium acquisition. Plant Physiol. 147, 252–262. 18359846 10.1104/pp.107.114546 PMC2330294

[pbi13332-bib-0025] Gajdanowicz, P. , Michard, E. , Sandmann, M. , Rocha, M. , Correa, L.G.G. , Ramirez‐Aguilar, S.J. , Gomez‐Porras, J.L. *et al.* (2011) Potassium (K^+^) gradients serve as a mobile energy source in plant vascular tissues. Proc. Natl Acad. Sci. USA, 108, 864–869. 21187374 10.1073/pnas.1009777108 PMC3021027

[pbi13332-bib-0026] Garcia‐Mata, C. , Gay, R. , Sokolovski, S. , Hills, A. , Lamattina, L. and Blatt, M.R. (2003) Nitric oxide regulates K^+^ and Cl^‐^ channels in guard cells through a subset of abscisic acid‐evoked signaling pathways. Proc. Natl Acad. Sci. USA, 100, 11116–11121. 12949257 10.1073/pnas.1434381100 PMC196936

[pbi13332-bib-0027] Gierth, M. , Maser, P. and Schroeder, J.I. (2005) The potassium transporter *AtHAK5* functions in K^+^ deprivation‐induced high‐affinity K^+^ uptake and *AKT1* K^+^ channel contribution to K^+^ uptake kinetics in *Arabidopsis* roots. Plant Physiol. 137, 1105–1114. 15734909 10.1104/pp.104.057216 PMC1065410

[pbi13332-bib-0028] Grabov, A. (2007) Plant KT/KUP/HAK potassium transporters: single family‐ multiple functions. Ann. Bot. 99, 1035–1041. 17495982 10.1093/aob/mcm066 PMC3243584

[pbi13332-bib-0029] Grefen, C. , Chen, Z. , Honsbein, A. , Donald, N. , Hills, A. and Blatt, M.R. (2010) A novel motif essential for snare interaction with the K^+^ channel KC1 and channel gating in *Arabidopsis* . Plant Cell, 22, 3076–3092. 20884800 10.1105/tpc.110.077768 PMC2965544

[pbi13332-bib-0030] He, X. , Zeng, J. , Cao, F. , Ahmed, I.M. , Zhang, G. , Vincze, E. and Wu, F. (2015) HvEXPB7, a novel beta‐expansin gene revealed by the root hair transcriptome of Tibetan wild barley, improves root hair growth under drought stress. J. Exp. Bot. 66, 7405–7419. 26417018 10.1093/jxb/erv436 PMC4765802

[pbi13332-bib-0031] Heckman, D.S. , Geiser, D.M. , Eidell, B.R. , Stauffer, R.L. , Kardos, N.L. and Hedges, S.B. (2001) Molecular evidence for the early colonization of land by fungi and plants. Science, 293, 1129–1133. 11498589 10.1126/science.1061457

[pbi13332-bib-0032] Held, K. , Pascaud, F. , Eckert, C. , Gajdanowicz, P. , Hashimoto, K. , Corratge‐Faillie, C. , Offenborn, J.N. *et al.* (2011) Calcium‐dependent modulation and plasma membrane targeting of the AKT2 potassium channel by the CBL4/CIPK6 calcium sensor/protein kinase complex. Cell Res. 21, 1116–1130. 21445098 10.1038/cr.2011.50 PMC3193494

[pbi13332-bib-0033] Hills, A. , Chen, Z.H. , Amtmann, A. , Blatt, M.R. and Lew, V.L. (2012) OnGuard, a computational platform for quantitative kinetic modeling of guard cell physiology. Plant Physiol. 159, 1026–1042. 22635116 10.1104/pp.112.197244 PMC3387691

[pbi13332-bib-0034] Hosy, E. , Vavasseur, A. , Mouline, K. , Dreyer, I. , Gaymard, F. , Poree, F. , Boucherez, J. *et al.* (2003) The *Arabidopsis* outward K^+^ channel GORK is involved in regulation of stomatal movements and plant transpiration. Proc. Natl Acad. Sci. USA, 100, 5549–5554. 12671068 10.1073/pnas.0733970100 PMC154382

[pbi13332-bib-0035] Huang, Y. , Cao, H. , Yang, L. , Chen, C. , Shabala, L. , Xiong, M. , Niu, M. *et al.* (2019) Tissue‐specific respiratory burst oxidase homolog‐dependent H_2_O_2_ signaling to the plasma membrane H^+^‐ATPase confers potassium uptake and salinity tolerance in Cucurbitaceae. J. Exp. Bot. 70, 5879–5893. 31290978 10.1093/jxb/erz328 PMC6812723

[pbi13332-bib-0036] Ivashikina, N. , Deeken, R. , Fischer, S. , Ache, P. and Hedrich, R. (2005) AKT2/3 subunits render guard cell K^+^ channels Ca^2+^ sensitive. J. Gen Physiol. 125, 483–492. 15824192 10.1085/jgp.200409211 PMC2217505

[pbi13332-bib-0037] Jan, L.Y. and Jan, Y.N. (1997) Cloned potassium channels from eukaryotes and prokaryotes. Annu. Rev. Neurosci. 20, 91–123. 9056709 10.1146/annurev.neuro.20.1.91

[pbi13332-bib-0038] Jegla, T. , Busey, G. and Assmann, S.M. (2018) Evolution and structural characteristics of plant voltage‐gated K^+^ channels. Plant Cell, 30, 2898–2909. 30389753 10.1105/tpc.18.00523 PMC6354262

[pbi13332-bib-0039] Lacombe, B. , Pilot, G. , Michard, E. , Gaymard, F. , Sentenac, H. and Thibaud, J.B. (2000) A shaker‐like K^+^ channel with weak rectification is expressed in both source and sink phloem tissues of *Arabidopsis* . Plant Cell, 12, 837–851. 10852932 10.1105/tpc.12.6.837 PMC149088

[pbi13332-bib-0040] Langer, K. , Ache, P. , Geiger, D. , Stinzing, A. , Arend, M. , Wind, C. , Regan, S. *et al.* (2002) Poplar potassium transporters capable of controlling K^+^ homeostasis and K^+^‐dependent xylogenesis. Plant J. 32, 997–1009. 12492841 10.1046/j.1365-313x.2002.01487.x

[pbi13332-bib-0041] Latz, A. , Ivashikina, N. , Fischer, S. , Ache, P. , Sano, T. , Becker, D. , Deeken, R. *et al.* (2007) In planta AKT2 subunits constitute a pH‐ and Ca^2+^‐sensitive inward rectifying K^+^ channel. Planta, 225, 1179–1191. 17146665 10.1007/s00425-006-0428-4

[pbi13332-bib-0042] Leebens‐Mack, J.H. , Barker, M.S. , Carpenter, E.J. , Deyholos, M.K. , Gitzendanner, M.A. *et al.* (2019) One thousand plant transcriptomes and the phylogenomics of green plants. Nature, 574, 679–685. 31645766 10.1038/s41586-019-1693-2 PMC6872490

[pbi13332-bib-0043] Li, J. , Li, Y. , Yin, Z. , Jiang, J. , Zhang, M. , Guo, X. , Ye, Z. *et al.* (2017) *OsASR5* enhances drought tolerance through a stomatal closure pathway associated with ABA and H_2_O_2_ signaling in rice. Plant Biotechnol. J. 15, 183–196. 27420922 10.1111/pbi.12601 PMC5258865

[pbi13332-bib-0044] Ma, L. , Zhang, H. , Sun, L. , Jiao, Y. , Zhang, G. , Miao, C. and Hao, F. (2012) NADPH oxidase AtrbohD and AtrbohF function in ROS‐dependent regulation of Na^+^/K^+^ homeostasis in *Arabidopsis* under salt stress. J. Exp. Bot. 63, 305–317. 21984648 10.1093/jxb/err280

[pbi13332-bib-0045] Maathuis, F.J. and Sanders, D. (1994) Mechanism of high‐affinity potassium uptake in roots of *Arabidopsis thaliana* . Proc. Natl Acad. Sci. USA, 91, 9272–9276. 7937754 10.1073/pnas.91.20.9272 PMC44794

[pbi13332-bib-0046] Mak, M. , Babla, M. , Xu, S.C. , O'Carrigan, A. , Liu, X.H. , Gong, Y.M. , Holford, P. *et al.* (2014) Leaf mesophyll K^+^, H^+^ and Ca^2+^ fluxes are involved in drought‐induced decrease in photosynthesis and stomatal closure in soybean. Environ. Exp. Bot. 98, 1–12.

[pbi13332-bib-0047] Marschner, H. (2012) Marschner's Mineral Nutrition of Higher Plants.San Diego, CA:Academic press.

[pbi13332-bib-0048] Michard, E. , Dreyer, I. , Lacombe, B. , Sentenac, H. and Thibaud, J.B. (2005) Inward rectification of the AKT2 channel abolished by voltage‐dependent phosphorylation. Plant J. 44, 783–797. 16297070 10.1111/j.1365-313X.2005.02566.x

[pbi13332-bib-0049] Newman, I.A. (2001) Ion transport in roots: measurement of fluxes using ion‐selective microelectrodes to characterize transporter function. Plant Cell Environ. 24, 1–14. 11762438 10.1046/j.1365-3040.2001.00661.x

[pbi13332-bib-0050] Palmgren, M.G. (2001) Plant plasma membrane H^+^‐ATPases: powerhouses for nutrient uptake. Annu. Rev. Plant Biol. 52, 817–845. 10.1146/annurev.arplant.52.1.817 11337417

[pbi13332-bib-0051] Pornsiriwong, W. , Estavillo, G.M. , Chan, K.X. , Tee, E.E. , Ganguly, D. , Crisp, P.A. , Phua, S.Y. *et al.* (2017) A chloroplast retrograde signal, 3′‐phosphoadenosine 5′‐phosphate, acts as a secondary messenger in abscisic acid signaling in stomatal closure and germination. eLife, 6, e23361. 28323614 10.7554/eLife.23361 PMC5406205

[pbi13332-bib-0052] Qiu, Y.‐L. and Palmer, J.D. (1999) Phylogeny of early land plants: insights from genes and genomes. Trends Plant Sci. 4, 26–29. 10234267 10.1016/s1360-1385(98)01361-2

[pbi13332-bib-0053] Redwan, M. , Spinelli, F. , Marti, L. , Weiland, M. , Palm, E. , Azzarello, E. and Mancuso, S. (2016) Potassium fluxes and reactive oxygen species production as potential indicators of salt tolerance in *Cucumis sativus* . Funct. Plant Biol. 43, 1016–1027. 32480523 10.1071/FP16120

[pbi13332-bib-0054] Regenberg, B. , Villalba, J.M. , Lanfermeijer, F.C. and Palmgren, M.G. (1995) C‐terminal deletion analysis of plant plasma membrane H^+^‐ATPase: yeast as a model system for solute transport across the plant plasma membrane. Plant Cell, 7, 1655–1666. 7580256 10.1105/tpc.7.10.1655 PMC161027

[pbi13332-bib-0055] Riedelsberger, J. , Dreyer, I. and Gonzalez, W. (2015) Outward rectification of voltage‐gated K^+^ channels evolved at least twice in life history. PLoS ONE, 10, e0137600. 26356684 10.1371/journal.pone.0137600 PMC4565715

[pbi13332-bib-0056] Rodriguez‐Navarro, A. and Rubio, F. (2006) High‐affinity potassium and sodium transport systems in plants. J. Exp. Bot. 57, 1149–1160. 16449373 10.1093/jxb/erj068

[pbi13332-bib-0057] Santa‐Maria, G.E. , Rubio, F. , Dubcovsky, J. and Rodriguez‐Navarro, A. (1997) The HAK1 gene of barley is a member of a large gene family and encodes a high‐affinity potassium transporter. Plant Cell, 9, 2281–2289. 9437867 10.1105/tpc.9.12.2281 PMC157074

[pbi13332-bib-0058] Selvaraj, M.G. , Ishizaki, T. , Valencia, M. , Ogawa, S. , Dedicova, B. , Ogata, T. , Yoshiwara, K. *et al.* (2017) Overexpression of an *Arabidopsis thaliana* galactinol synthase gene improves drought tolerance in transgenic rice and increased grain yield in the field. Plant Biotechnol. J. 15, 1465–1477. 28378532 10.1111/pbi.12731 PMC5633756

[pbi13332-bib-0059] Shabala, S. and Cuin, T.A. (2008) Potassium transport and plant salt tolerance. Physiol. Plant. 133, 651–669. 18724408 10.1111/j.1399-3054.2007.01008.x

[pbi13332-bib-0060] Shabala, S.N. and Lew, R.R. (2002) Turgor regulation in osmotically stressed *Arabidopsis* epidermal root cells. Direct support for the role of inorganic ion uptake as revealed by concurrent flux and cell turgor measurements. Plant Physiol. 129, 290–299. 12011359 10.1104/pp.020005 PMC155892

[pbi13332-bib-0061] Shabala, S. and Pottosin, I. (2014) Regulation of potassium transport in plants under hostile conditions: implications for abiotic and biotic stress tolerance. Physiol. Plant. 151, 257–279. 24506225 10.1111/ppl.12165

[pbi13332-bib-0062] Shabala, L. , Zhang, J. , Pottosin, I. , Bose, J. , Zhu, M. , Fuglsang, A.T. , Velarde‐Buendia, A. *et al.* (2016) Cell‐type‐specific H^+^‐ATPase activity in root tissues enables K^+^ retention and mediates acclimation of barley (*Hordeum vulgare*) to salinity stress. Plant Physiol. 172, 2445–2458. 27770060 10.1104/pp.16.01347 PMC5129721

[pbi13332-bib-0063] Shabala, S. , Chen, G. , Chen, Z.H. and Pottosin, I. (2019) The energy cost of the tonoplast futile sodium leak. New Phytol. 225, 1105–1110. 30802968 10.1111/nph.15758

[pbi13332-bib-0064] Shi, H. , Ye, T. , Zhu, J. and Chan, Z. (2014) Constitutive production of nitric oxide leads to enhanced drought stress resistance and extensive transcriptional reprogramming in *Arabidopsis* . J. Exp. Bot. 65, 4119–4131. 24868034 10.1093/jxb/eru184 PMC4112625

[pbi13332-bib-0065] Shin, R. and Schachtman, D.P. (2004) Hydrogen peroxide mediates plant root cell response to nutrient deprivation. Proc. Natl Acad. Sci. USA, 101, 8827–8832. 15173595 10.1073/pnas.0401707101 PMC423280

[pbi13332-bib-0066] Sklodowski, K. , Riedelsberger, J. , Raddatz, N. , Riadi, G. , Caballero, J. , Chérel, I. , Schulze, W. *et al.* (2017) The receptor‐like pseudokinase MRH1 interacts with the voltage‐gated potassium channel AKT2. Sci. Rep. 7, 44611. 28300158 10.1038/srep44611 PMC5353636

[pbi13332-bib-0067] Umezawa, T. , Fujita, M. , Fujita, Y. , Yamaguchi‐Shinozaki, K. and Shinozaki, K. (2006) Engineering drought tolerance in plants: discovering and tailoring genes to unlock the future. Curr. Opin. Biotechnol. 17, 113–122. 16495045 10.1016/j.copbio.2006.02.002

[pbi13332-bib-0068] Véry, A.A. and Sentenac, H. (2003) Molecular mechanisms and regulation of K^+^ transport in higher plants. Annu. Rev. Plant Biol. 54, 575–603. 14503004 10.1146/annurev.arplant.54.031902.134831

[pbi13332-bib-0069] Wang, Y. and Wu, W.H. (2013) Potassium transport and signaling in higher plants. Annu. Rev. Plant Biol. 64, 451–476. 23330792 10.1146/annurev-arplant-050312-120153

[pbi13332-bib-0070] Wang, Y. , Noguchi, K. , Ono, N. , Inoue, S.I. , Terashima, I. and Kinoshita, T. (2014) Overexpression of plasma membrane H^+^‐ATPase in guard cells promotes light‐induced stomatal opening and enhances plant growth. Proc. Natl Acad. Sci. USA, 111, 533–538. 24367097 10.1073/pnas.1305438111 PMC3890815

[pbi13332-bib-0071] Wang, F. , Chen, Z.H. , Liu, X. , Colmer, T.D. , Shabala, L. , Salih, A. , Zhou, M. *et al.* (2017) Revealing the roles of GORK channels and NADPH oxidase in acclimation to hypoxia in *Arabidopsis* . J. Exp. Bot. 68, 3191–3204. 28338729 10.1093/jxb/erw378 PMC5853854

[pbi13332-bib-0072] Wang, X. , Chen, Z.H. , Yang, C. , Zhang, X. , Jin, G. , Chen, G. , Wang, Y. *et al.* (2018) Genomic adaptation to drought in wild barley is driven by edaphic natural selection at the Tabigha Evolution Slope. Proc. Natl Acad. Sci. USA, 115, 5223–5228. 29712833 10.1073/pnas.1721749115 PMC5960308

[pbi13332-bib-0073] Wu, D. , Shen, Q. , Qiu, L. , Han, Y. , Ye, L. , Jabeen, Z. , Shu, Q. *et al.* (2014) Identification of proteins associated with ion homeostasis and salt tolerance in barley. Proteomics, 14, 1381–1392. 24616274 10.1002/pmic.201300221

[pbi13332-bib-0074] Xicluna, J. , Lacombe, B. , Dreyer, I. , Alcon, C. , Jeanguenin, L. , Sentenac, H. , Thibaud, J.B. *et al.* (2007) Increased functional diversity of plant K^+^ channels by preferential heteromerization of the shaker‐like subunits AKT2 and KAT2. J. Biol. Chem. 282, 486–494. 17085433 10.1074/jbc.M607607200

[pbi13332-bib-0075] Ye, L. , Wang, Y. , Long, L. , Luo, H. , Shen, Q. , Broughton, S. , Wu, D. *et al.* (2019) A Trypsin family protein gene controls tillering and leaf shape in barley. Plant Physiol. 181, 701–713. 31427466 10.1104/pp.19.00717 PMC6776861

[pbi13332-bib-0076] Zeng, F. , Konnerup, D. , Shabala, L. , Zhou, M. , Colmer, T.D. , Zhang, G. and Shabala, S. (2014) Linking oxygen availability with membrane potential maintenance and K^+^ retention of barley roots: implications for waterlogging stress tolerance. Plant, Cell Environ. 37, 2325–2338. 25132404 10.1111/pce.12422

[pbi13332-bib-0077] Zhang, Y.D. , Véry, A.A. , Wang, L.M. , Deng, Y.W. , Sentenac, H. and Huang, D.F. (2011) A K^+^ channel from salt‐tolerant melon inhibited by Na^+^ . New Phytol. 189, 856–868. 21077888 10.1111/j.1469-8137.2010.03526.x

[pbi13332-bib-0078] Zhang, A. , Ren, H.M. , Tan, Y.Q. , Qi, G.N. , Yao, F.Y. , Wu, G.L. , Yang, L.W. *et al.* (2016) S‐type anion channels SLAC1 and SLAH3 function as essential negative regulators of inward K^+^ channels and stomatal opening in *Arabidopsis* . Plant Cell, 28, 949–965. 27002025 10.1105/tpc.15.01050 PMC4863386

[pbi13332-bib-0079] Zhang, X. , Wu, H. , Chen, L. , Wang, N. , Wei, C. and Wan, X. (2019) Mesophyll cells' ability to maintain potassium is correlated with drought tolerance in tea (*Camellia sinensis*). Plant Physiol. Biochem. 136, 196–203. 30685699 10.1016/j.plaphy.2019.01.020

[pbi13332-bib-0080] Zhao, C. , Wang, Y. , Chan, K.X. , Marchant, D.B. , Franks, P.J. , Randall, D. , Tee, E.E. *et al.* (2019) Evolution of chloroplast retrograde signaling facilitates green plant adaptation to land. Proc. Natl Acad. Sci. USA, 116, 5015–5020. 30804180 10.1073/pnas.1812092116 PMC6421419

