# Supplementary material for: HvAKT2 and HvHAK1 confer drought tolerance in barley through enhanced leaf mesophyll H+ homoeostasis
Source: Plant Biotechnol J. 2020 Jan 24;18(8):1683–96. doi: 10.1111/pbi.13332 (PMC7336388; doi:10.1111/pbi.13332)
Supplement: Supplementary file 1 — Figure S1 Phylogenetic analysis of AKT2 and HAK1 in different plant and algal species selected from the 1KP database. Figure S2 Protein evolutionary analysis of AKT2 and HAK1 using PIECE with an inbuilt GLOOME and an inbuilt Exalign function in plant and algal species. Figure S3 Functional domain of HvAKT2 and HvHAK1. Figure S4 Electrophysiology of HvAKT2 and HvHAK1 in Xenopus laevis oocytes. Figure S5 BSMV‐VIGS used in wild barley XZ5. Figure S6 Analysis of the expression of HvAKT2 and HvHAK1 in transgenic overexpression lines using qRT‐PCR. Figure S7 The K+ concentration in roots of silenced and overexpression lines. Figure S8 The activity of H+‐ATPase in roots of silenced and overexpression lines. Figure S9 Transient and steady‐state changes in K+, Ca2+ and H+ ﬂuxes from root epidermal cells of inoculated plants via BSMV‐VIGS subjected to 20% PEG treatments. Table S1 Statistics of evolution of AKT2s and HAK1s in the 1KP dataset Table S2 List of quantitative real‐time PCR primers Table S3 List of in situ PCR primers Table S4 List of primers for vector construction of BSMV:HvPDS, BSMV:HvAKT2 and BSMV:HvHAK1 Table S5 List of primers for heterologous expression in Xenopus laevis oocytes and barley transformation Table S6 Correlation analysis among all parameters in different barley lines in the control and drought [file PBI-18-1683-s001.docx]

**Fig. S1**. Phylogenetic analysis of AKT2 and HAK1 in different plant and algal species selected from the 1KP database. All the transcriptomes can be accessed from the One Thousand Plants Project. Candidate protein sequences were acquired by BLASTP searches using HvAKT2 and HvHAK1 as the query with the criterion of E-value<10^-5^.

**Fig. S2. A,** Protein evolutionary analysis of AKT2 using PIECE (http://www.bioinfogenome.net/piece/) with an inbuilt GLOOME function (http://www.bioinfogenome.net/piece/gloome.php) in plant and algal species.


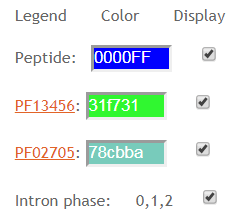


**Fig. S2. B,** Protein evolutionary analysis of HAK1 using PIECE (http://www.bioinfogenome.net/piece/) with an inbuilt GLOOME function (http://www.bioinfogenome.net/piece/gloome.php) in plant and algal species.

**Fig. S2. C,** Gene evolutionary analysis of AKT2 using PIECE (<http://www.bioinfogenome.net/piece/>) with an inbuilt Exalign (http://www.bioinfogenome.net/piece/exalign.php/) in plant and algal species.


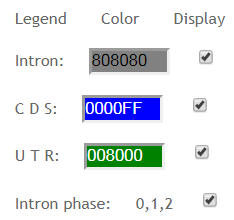


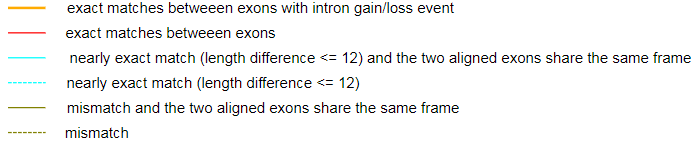


**Fig. S2. D,** Gene evolutionary analysis of HAK1 using PIECE (<http://www.bioinfogenome.net/piece/>) with an inbuilt Exalign (http://www.bioinfogenome.net/piece/exalign.php/) in plant and algal species.

**(a)**


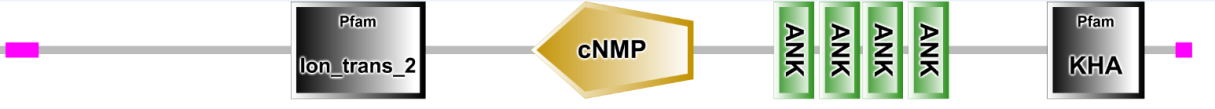


100

200

300

400

500

600

0

800

700

**(b)**

100

200

300

400

500

600

0

700


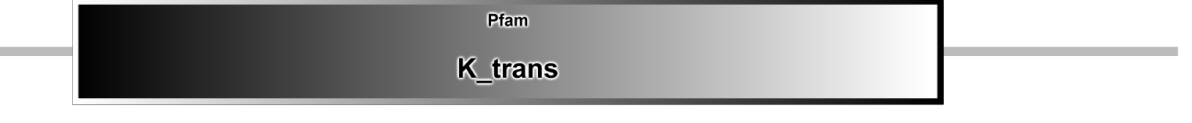


**Fig. S3.** Functional domains of HvAKT2 (a) and HvHAK1 (b). Pink color represents low complexity sequence.

**Fig. S4.** Electrophysiology of HvAKT2 and HvHAK1 in *Xenopus laevis* oocytes. Effect of K^+^ on HvAKT2- and HvHAK1-mediated currents in *Xenopus laevis* oocytes. Water (control) means water-injected oocytes. No currents were detected in water-injected oocytes at different K^+^ levels. The holding potential was -20 mV and the voltage was clamped from -160 to 0 mV for 10 cycles. Data are means ± SDs (10–15 oocytes from at least three independent experiments).

**(b)**

**(a)**

*Actin*

*HvPDS*


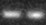

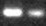


Control


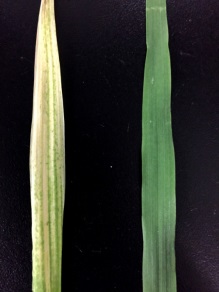


BSMV:HvPDS

BSMV:HvPDS

Control

**Fig. S5.** BSMV-VIGS used in wild barley XZ5. (a) Photobleaching of BSMV:HvPDS-inoculated plants in contrast to those treated with empty vectors. (b) qRT-PCR analysis of *HvPDS* transcript abundance in leaves of XZ5.

**Fig. S6** Analysis of the expression of *HvAKT2* and *HvHAK1* in transgenic overexpression lines using qRT-PCR. HvAKT2-OX2 and HvHAK1-OX2 lines were used for functional verification. Data are means ± SDs (n=6).

**Fig. S7** The K content in roots of silenced and overexpression lines. Seedlings were grown in BNS for 10 d, and then treated by PEG 6000 for 5 d. Error bars represent SD values (n=6), different letters indicate significant differences (*P<0.05*). Control and drought correspond to basic nutrition solution (BNS), BNS+20% PEG, respectively.

**Fig. S8** The activity of H^+^-ATPase in roots of silenced (a) and overexpression (b) lines. Seedlings were grown in BNS for 10 d, and then treated by PEG 6000 for 5 d. Error bars represent SD values (n=6), different letters indicate significant differences (*P<0.05*). Control and drought correspond to basic nutrition solution (BNS), BNS+20% PEG, respectively.

**Fig. S9** Transient (a, c, e) and steady-state (b, d, f) changes in K^+^, Ca^2+^ and H^+^ ﬂuxes from root epidermal cells of inoculated plants *via* BSMV-VIGS subjected to 20% PEG treatments. Roots were sampled from BSMV:γ, BSMV:HvAKT2 and BSMV:HvHAK1 -inoculated plants grown in BNS for 10 d. Roots were pre-incubated in the BSM (0.5 mMKCl and 0.1 mM CaCl_2_) for 2 h. For transient ion fluxes, data are means ± SE (n = 8). Steady-state ion ﬂuxes were measured over 0, 1, 12 and 24h on PEG exposure. Data are means ± SE (n = 10)

**Table S1.** Statistics of evolution of AKT2s and HAK1s in the 1KP dataset.

| order | Total No, of species | AKT2 in No, of species | HAK1 in No, of species | AKT2,% | HAK1,% | TXXTXGYGD in AKT2, No. of species |
| --- | --- | --- | --- | --- | --- | --- |
| Angiosperms | 830 | 710 | 675 | 86 | 81 | 622 |
| Gymnosperms | 84 | 72 | 72 | 86 | 86 | 70 |
| Ferns | 74 | 57 | 64 | 77 | 86 | 26 |
| Lycophytes | 22 | 10 | 21 | 45 | 95 | 6 |
| Mosses | 50 | 40 | 45 | 80 | 90 | 37 |
| Liverworts | 28 | 24 | 26 | 86 | 93 | 21 |
| Streptophyte algae | 54 | 40 | 35 | 74 | 65 | 26 |
| Chlorophyte algae | 117 | 81 | 46 | 69 | 39 | 28 |
| Rhodophyta | 28 | 16 | 2 | 57 | 7 | 15 |
| Chromista | 35 | 5 | 3 | 14 | 9 | 7 |
| Total/Average | 1322 | 1055 | 989 | 80 | 75 | 858 |

*Note*: Candidate protein sequences were selected by BLASTP searches using Arabidopsis sequences as the query with the criterion of E-value<10^-5^ in the 1KP database.

**Table S2.** List of quantitative real-time PCR primers

| **Name of Primers Designed** | **Forward Primer** | **Genbank** | **Reverse Primer** | **Amplicon**  **Size (bp)** | |
| --- | --- | --- | --- | --- | --- |
| *RT-HvHAK1* | ATAGGCGATGGAACCTTGAC | AF025292.2 | GACCGAGAAGAGCATGAACA | | 135 |
| *RT-HvAKT2* | CTCAAGTTCAGGGCGGAGAG | DQ465923.1 | TCCTCCTTGGGCGGTATGTA | | 194 |
| *RT-HvHA1* | TGTTGTTGTTGGCTGCAAGG | AJ344078.1 | TGCCAGTTACCCTCAGCATC | | 184 |
| *HvActin*  *(reference gene)* | TGGCTGACGGTGAGGACA | AY145451.1 | CGAGGGCGACCAACTATG | | 121 |

**Table S3.** List of *in situ* PCR primers

| **Name of Primers Designed** | **Forward Primer** | **Reverse Primer** |  |
| --- | --- | --- | --- |
| *ISRT-HvHAK1* | TCAGACACAAGTGGTCCTCA | TGGTGCTGGGATGGATCTAT | |
| *ISRT-HvAKT2* | CCAGATACAGGTGCTGGGAG | CCAGCCTTGTGAAGAATTGC | |
| *IS-HvActin*  *(reference gene)* | AATGGTCAAGGCTGGTTTCG | AGAACGATACCAGTAGTACG | |

**Table S4**. List of primers for vector construction of BSMV:HvPDS, BSMV:HvAKT2 and BSMV:HvHAK1

| **Name of Primers Designed** | **Primer** | **Amplicon Size (bp)** |
| --- | --- | --- |
| *PDS-*γ-F | GTACGCTAGCCGACGAGGTTTTTATTGC | 286bp |
| *PDS-*γ-R | GTACGCTAGCAGTTATTTGAGTCCCGTC |  |
| *AKT2-*γ-F | GTACGCTAGCTACATACCGCCCAAGGAGGA | 383bp |
| *AKT2-*γ-R | GTACGCTAGCAGATTGCACGGGACGATGTT |  |
| *HAK1-*γ-F | GTACGCTAGCATAGGCGATGGAACCTTGAC | 326bp |
| *HAK1-*γ-R | GTACGCTAGCCCACCAAGTGAGACCCATCC |  |
| γ-stain-F | CAACTGCCAATCGTGAGTAGG |  |

| **Name of Primers Designed** | **Primer** |
| --- | --- |
| *attB-HvAKT2-F* | GGGGACAAGTTTGTACAAAAAAGCAGGCTTAATGAAGATCCCCAGCTTC |
| *attB-HvAKT2-R* | GGGGACCACTTTGTACAAGAAAGCTGGGTTTATATGACACTTGCTACT |
| *attB-HvHAK1-F* | GGGGACAAGTTTGTACAAAAAAGCAGGCTTAATGTCGCTGCAAGTCGAG |
| *attB-HvHAK1-R* | GGGGACCACTTTGTACAAGAAAGCTGGGTCTATATCTCGTATGTGAT |

**Table S5**. List of primers for heterologous expression in *Xenopus laevis* oocytes and barley transformation.

**Table S6**. Correlation analysis among all parameters in different barley lines in the control and drought.

| Parameters | NO  content | H_2_O_2_ content | Biomass | K concentration | H^+^-ATPase activity | K^+^ fluxes | Ca^2+^ fluxes | H^+^ fluxes | *HvAKT2* expression | *HvHAK1* expression | *HvHA1* expression |
| --- | --- | --- | --- | --- | --- | --- | --- | --- | --- | --- | --- |
| NO content | 1 |  |  |  |  |  |  |  |  |  |  |
| H_2_O_2_ content | 0.325 | 1 |  |  |  |  |  |  |  |  |  |
| Biomass | 0.009 | 0.019 | 1 |  |  |  |  |  |  |  |  |
| K concentration | 0.868** | 0.358 | 0.002 | 1 |  |  |  |  |  |  |  |
| H^+^-ATPase activity | 0.826** | 0.184 | 0.064 | 0.694* | 1 |  |  |  |  |  |  |
| K^+^ fluxes | 0.547 | 8E-06 | 0.255 | 0.427 | 0.644* | 1 |  |  |  |  |  |
| Ca^2+^ fluxes | 0.039 | 0.005 | 0.320 | 0.023 | 0.047 | 0.084 | 1 |  |  |  |  |
| H^+^ fluxes | 0.025 | 0.646* | 0.240 | 0.029 | 0.001 | 0.178 | 0.195 | 1 |  |  |  |
| *HvAKT2* expression | 0.666* | 0.162 | 0.094 | 0.786** | 0.488 | 0.508 | 0.121 | 0.006 | 1 |  |  |
| *HvHAK1* expression | 0.499 | 0.268 | 0.126 | 0.588* | 0.602* | 0.405 | 0.060 | 0.002 | - | 1 |  |
| *HvHA1* expression | 0.898** | 0.227 | 0.069 | 0.845** | 0.830** | 0.621* | 0.058 | 0.001 | 0.708** | 0.554* | 1 |

*Note*: Average value of each parameter was used for correlation analysis. *P<0.05; **P<0.01.
